# Expression of a novel brain specific isoform of C3G is regulated during development

**DOI:** 10.1038/s41598-020-75813-z

**Published:** 2020-11-02

**Authors:** Divya Sriram, Ramulu Chintala, B. V. V. Parthasaradhi, Sanjeev Chavan Nayak, Indumathi Mariappan, Vegesna Radha

**Affiliations:** 1 CSIR-Centre for Cellular and Molecular Biology, Uppal Road, Hyderabad, 500 007 India; 2 Sudhakar and Sreekanth Ravi Stem Cell Biology Laboratory, Prof Brien Holden Eye Research Centre, Hyderabad Eye Research Foundation, L.V. Prasad Eye Institute, Hyderabad, India

**Keywords:** Cell biology, Developmental biology

## Abstract

Mice lacking C3G (RapGEF1), a ubiquitously expressed protein essential for neuronal differentiation, show multiple defects in brain development. Function of C3G in neurogenesis is poorly defined. Here, we identify brain specific expression of a novel C3G isoform in mice and humans. This isoform has an insert in the Crk-binding region, generating a polypeptide of 175 kDa, unlike the previously known 140 kDa form expressed in all other tissues. In the adult mouse brain, C3G expression is seen in neurons, but was not detectable in GFAP-positive cells. C3G levels were high in the CA3 region of hippocampus and in mitral cells of olfactory bulb. Neural progenitor cells positive for Doublecortin and Nestin, show expression of C3G. During development, C3G is expressed in precursor cells prior to their differentiation into mature neurons or astrocytes. The 175 kDa as well as 140 kDa forms are seen in embryonic mouse brain, while only the 175 kDa variant is seen in post-natal brain. Human cerebral organoids generated from induced pluripotent stem cells predominantly expressed the 140 kDa polypeptides, and the 175 kDa isoform appeared upon maturation. This study describes developmental regulation and neuronal expression of a brain specific isoform of C3G, a molecule essential for normal development of the mammalian brain.

## Introduction

Guanine Nucleotide Exchange Factors (GEFs) play an important role in regulating cellular functions by acting as hubs in pathways to transmit extracellular cues to alter gene expression and remodel the cytoskeleton^1,2^. GEFs generally activate specific classes of GTPases, to engage distinct effectors. C3G (RapGEF1) is a ubiquitously expressed molecule that targets Ras family members, Rap1 and R-Ras, as well as Rho family GTPase, TC10^3–6^. In all mammalian tissues and cell lines examined, it is a 140 kDa protein with a catalytic domain at the C-terminal and a central region containing multiple poly-proline tracts called Crk-Binding Region (CBR). It is through this domain that C3G interacts with a large number of proteins containing SH3 domain such as c-Abl, Crk, Hck and Cas^7–11^ as well as those that do not contain SH3 domain like β-catenin and TC48^12–14^. Species and tissue-specific alternately spliced forms of C3G that primarily differ at the N-terminal, and in the CBR, have been reported^11,15–17^. C3G localization and activity is regulated by protein interaction and phosphorylation^9,18–21^. It is predominantly localized to the cytoplasm, but undergoes regulated nuclear translocation^22^.

The function of C3G in signaling is dependent on its catalytic domain, protein interaction domain or both^9,23–27^. Its role in proliferation, cytoskeletal re-organization, transformation, survival and differentiation have been described^2^. C3G is essential for differentiation of neuronal, megakaryocyte, and muscle cells^28–30^. Nerve Growth Factor (NGF) induces complex formation of C3G with Tropomyosin Receptor Kinase A in endosomes and Rap1 activation in neuronal cells^31^. We have earlier shown that NGF or forskolin stimulation of neuroblastoma cells results in tyrosine phosphorylation and activation of C3G at the Golgi. Dephosphorylation by a Golgi-localized tyrosine phosphatase results in inhibition of neuronal differentiation^14,29^. C3G is essential for early embryonic development in mammals^32^. Mice compromised for normal C3G levels exhibit multiple defects in nervous system development^33–36^. C3G is engaged in response to Reelin, a factor important for neuronal differentiation and positioning^35,37–39^. C3G is also required for bipolar axon formation from multi-polar cells^36^. To study tissue-specific effects of loss of C3G, mice with expression of hypomorphic alleles (C3G^gt/gt^) were examined and found to exhibit defects in vascular system and died by 11.5 dpc. These mice showed defects in cortical neuroepithelial development with excessive proliferation of cortical neurons without differentiation. The embryos were defective in migration of sympathetic pre-ganglionic neurons in the spinal cord^33,35,39,40^.

During development, the mammalian brain undergoes lineage specific differentiation of precursor cells. Cells migrate, and are patterned to establish specialized regions, with distinct cell types and functions. These processes are recapitulated in in vitro grown brain organoids^41–47^. Oligodendrocytes, astrocytes, microglia and neurons make up the brain, and prominent areas in the adult brain are the cortex, olfactory lobes, hippocampus, thalamus, hypothalamus, cerebellum and medulla. Adult brain neurogenesis occurs in spatially restricted areas like the sub-ventricular zone, dentate gyrus and olfactory bulb. Neural progenitor cells (NPCs) can be identified by the presence of distinct marker proteins like Nestin and Doublecortin (DCX)^48,49^. Differentiation and specialization during neural fate specification are achieved by expression of distinct mRNAs and proteins, and cytoskeletal reorganization^50–55^. As a primary requirement for understanding role of C3G in development of the brain and nervous system in mammals, we examined the spatio-temporal expression and regulation of C3G in the brain. Using mouse and human tissue, we identify brain specific expression of an alternate isoform of C3G, which generates a polypeptide of about 175 kDa. Examination of C3G expression in developing mouse brain and human cerebral organoids showed developmental regulation of the C3G isoforms.

## Results

### C3G expression in human and mouse brain

**Figure 1:**
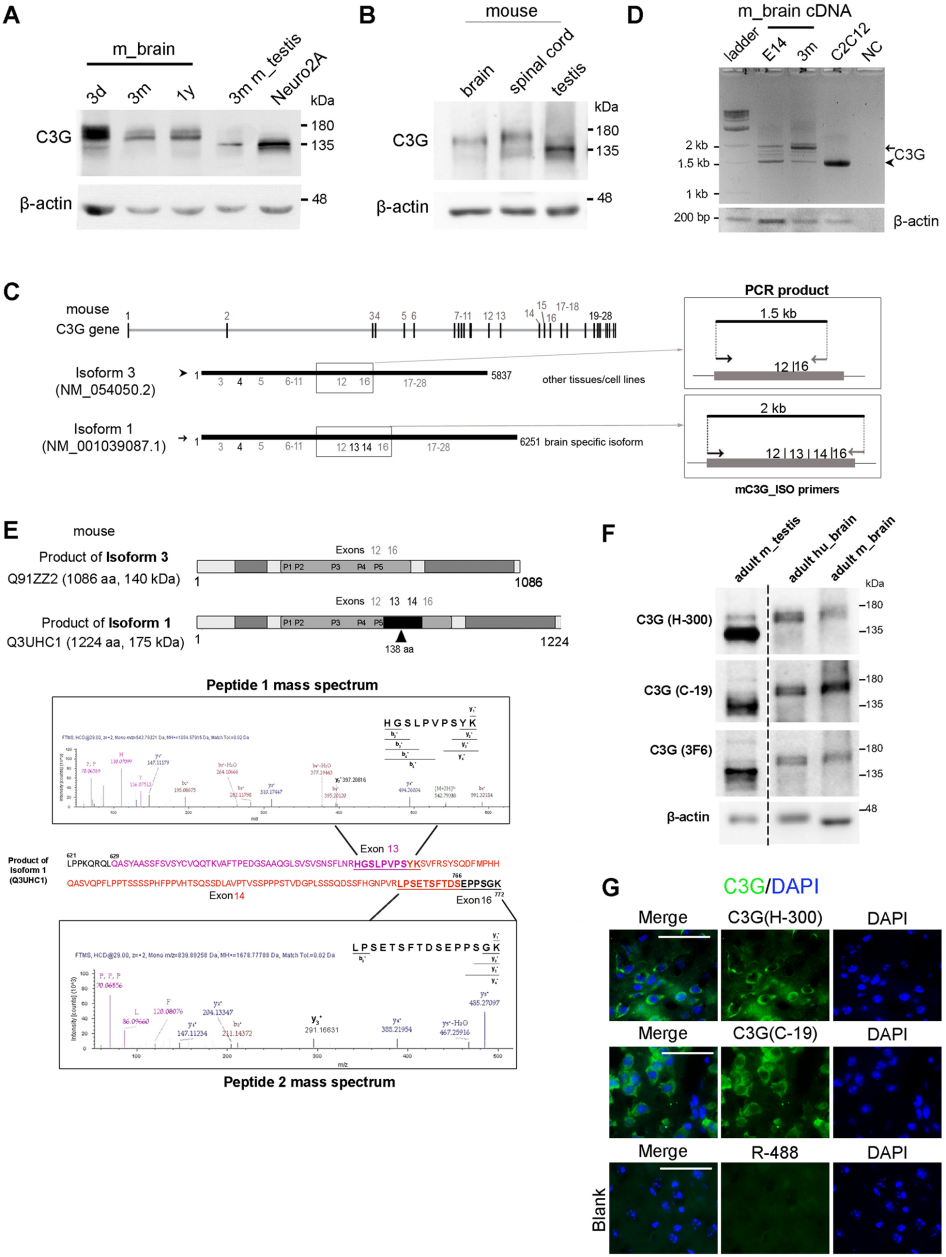
Expression of C3G in mouse brain tissue. (**A**) Lysates of brains obtained from 3-days-old (3d), 3-months-old (3 m) and 1-year-old (1y) mice were subjected to western blotting and probed with C3G (H300) antibody to detect C3G. Lysates of testis tissue from 3m-old mouse and Neuro2A cells were also used for comparison. After probing for C3G, the lower part of the blot was cut between 63 and 48 kDa markers, and probed for β-actin as a loading control. (**B**) Expression of C3G in spinal cord. Western blotting of tissues obtained from 3m-old mouse brain, spinal cord and testis were probed with H300 antibody to detect C3G. β-Actin was used as loading control. (**C**) Schematic representation of mouse C3G gene and exons. Alternately spliced transcripts, mouse isoforms 3 and 1 with their NCBI-Nucleotide ID’s are shown below. Exon numbers are indicted below the transcripts. The longer isoforms have additional exons (13, 14) in the region that encodes the CBR domain of C3G protein (shown in the boxed region). m_C3G_ISO primers were designed to give products of different lengths based on presence or absence of exons 12–15. Expected products from isoforms 1 and 3 are shown, along with their sizes. (**D**) Isoform specific amplification of C3G transcripts. RNA was extracted and cDNA prepared from 14-day mouse embryo brain (E14) and 3-month-old mouse brain tissue. Semi-quantitative PCR was performed using primers designed to differentiate between the C3G isoforms. cDNA prepared from C2C12 cells was used as a control for expression of NM_054050.2 isoform. β-Actin was used as internal control. NC indicates no-template control for the PCR. Arrow and arrowhead indicate the 2 kb and 1.5 kb PCR products, respectively. (**E**) Identification of peptides from C3G expressed in brain by mass spectrometry. Schematic shows the domains present in the 140 kDa and 175 kDa isoforms of C3G. Position of the exons, poly-proline tracts (P1–P5) and residues from the additional exon (black box) are indicated. The 175 kDa polypeptide of C3G immunoprecipitated from adult mouse brain was subjected to mass spectrometry. Identified peptides (underlined) corresponding to sequences present in the 138 amino acid insert (pink letters) of the longer isoform are shown. (**F**) Expression of C3G in adult human brain tissue. Lysates from adult mouse testis, human brain and adult mouse brain were subjected to western blotting using three C3G antibodies as indicated. The upper part of the blot was cut at the 100 kDa marker to probe for C3G. The blot was deprobed after probing with each of the antibodies. β-Actin was used as a loading control. The vertical dotted line indicates removal of three intervening lanes. (**G**) C3G localization in mouse brain cortex. Immunohistochemistry of cortical sections was carried out using the two indicated antibodies raised against non-overlapping domains of C3G. Images shown were captured using the Axioimager Z1 microscope. Blank image shows section processed similarly without addition of primary antibody and only the anti-rabbit Alexa-488 (R-488) secondary antibody. Scale bar, 50 µm.

Studies from our lab as well as that of others have shown the expression of C3G RNA and protein in a variety of tissues and cell types^15,16,28,38,56,57^. Prior to examining the expression and localization of C3G in the mouse brain by immunohistochemistry, we analyzed C3G expression in brain tissues obtained from mice of different age groups by western blotting. Whole brain lysates showed the presence of two closely running polypeptides of molecular weight around 175 kDa when examined using commercial polyclonal antibodies, H300, and C-19, that recognize the N-terminal and C-terminal of C3G, respectively (Fig. 1A, Supplementary figure 1A). Their specificity to detect C3G in mouse brain lysate by western blot is shown in Supplementary figure 1A. These antibodies have been used extensively to study cellular C3G and specifically recognize C3G, and no other proteins from cell lysates^10,13,22,25,27,31,38,39,57–66^. As an alternate tissue type and cell line for comparison, lysates prepared from 3-months-old mouse testis and Neuro2A cells were used, which showed C3G-specific polypeptides only at 140 kDa (Fig. 1A and Supplementary figure 1C) as described earlier^28,29,57^. Mouse spinal cord also showed expression of higher molecular weight polypeptides similar to that of the brain when probed using C3G H300 or C-19 antibodies (Fig. 1B and Supplementary figure 1B). The expression of the higher molecular weight C3G polypeptides in the brain, was validated using an alternate monoclonal antibody, 3F6mAb, that recognizes epitopes in the CBR. This antibody recognizes 140 kDa polypeptides of C3G in a variety of cell lines^57^, but detected 175 kDa polypeptide in the brain (Supplementary figure 1C). Therefore, it appears that mouse brain expresses alternate isoforms of C3G that give rise to larger polypeptides of molecular weight 175 kDa, unlike the 140 kDa polypeptide, which is seen in other tissues and cell types.

The mouse C3G gene has 28 exons, and the widely expressed 140 kDa protein arises from a 5837 bp C3G transcript, described as mouse C3G isoform 3 (NM_054050.2), which has 24 exons (Fig. 1C). RefSeq database search showed the presence of two longer isoforms of C3G that could produce polypeptides of 175 kDa-C3G isoform 1(NM_001039087.1; 6251 bp), and isoform 4, (NM_001362702.1; 6134 bp) obtained from a cDNA library made from adult mouse brain (FANTOM Consortium, RIKEN Genome Exploration Research Group). These isoforms 1 and 4, differed from the shorter isoform 3 (NM_054050.2), in having additional exons 13 and 14, in the CBR coding sequence (Supplementary figure 2A)^67,68^. Exons of other sizes have also been seen at the same splice site of C3G^68,69^ (mouse C3G isoform 2, NM_001039086.1 found in adult mouse testis cDNA RIKEN library and RIKEN full-length enriched mouse cDNA library from pooled tissues). Isoform 2 has an insert of 511 bp, corresponding to exons 13, 14 and 15 (Supplementary figure 2C). Isoforms 2 and 4 differ from isoforms 1 and 3 at the N-terminal (Supplementary figure 2A).

The size of the predicted polypeptide of the longer C3G isoforms, corresponded to that of the larger polypeptide (175 kDa) expressed in mouse brain. In order to determine which of the longer isoforms was expressed in the mouse brain, we prepared cDNA to carry out PCR with specific primers. Primers positioned on either side of the exon positions 12 and 16 were designed to give rise to amplicons of different lengths based on presence or absence of exons 13, 14 and 15 (Fig. 1C, Supplementary figure 2C). While a product of about 1.5 kb was amplified from C2C12 cells, brain tissue from embryonic-day 14, and adult (3-months-old) mouse, showed a major amplicon of about 2 kb (Fig. 1D). The 2 kb and 1.5 kb amplicons were sequenced using m_C3G_ISO forward and reverse primers. The 2 kb fragment showed the presence of nucleotides seen in the longer isoforms from the brain cDNA library that matched the transcripts having additional exons 13 and 14 (Fig. 1C, Supplementary figure 3A). In the protein sequence, this insertion is located at an exon junction in the sequence following the residues that code for the fifth poly-proline tract in CBR (Fig. 1E). Sequencing using reverse primer, showed that the 2 kb amplicon lacks the extra base pairs seen in isoform 4 with exon 15 (data not shown). The 1.5 kb insert lacked any of these residues and had contiguous sequence corresponding to isoform 3, with exon 12 spliced to exon 16 (Supplementary figure 3B). To determine the presence of exon 4 in the major brain transcript, we used m_N-term primers, specific to this region, which would give rise to a 550 bp product if exon 4 was present (Supplementary figure 2B). Thus, we confirmed that the brain specific isoform was C3G isoform 1, as it has exons 4, 13 and 14 apart from those seen in Isoform 3.

Interestingly, we observed differential expression of isoforms 1 (175 kDa) and 3 (140 kDa) in brain tissue derived from the embryo, and adult mouse. Level of the longer transcript (isoform 1) was higher in the adult brain, while the shorter transcript (isoform 3) was higher in the embryonic brain (Fig. 1D). The predominantly expressed isoform 1 in mouse brain tissue, therefore, could be giving rise to larger polypeptides of 175 kDa seen in western blotting. This was confirmed by carrying out mass spectrometric analysis of C3G protein immunoprecipitated from adult mouse brain. The 175 kDa polypeptide analyzed is shown in Supplementary figure 2E. Analysis showed the presence of two peptides, with sequence that corresponds to fragments translated from exons 13 and 14 of the mouse C3G isoform 1(Fig. 1E). The absence of exon 15 in this isoform was evidenced by the presence of a peptide with sequence generated from exon 14 spliced to exon 16 (peptide 2 in Fig. 1E). Amplicons of sizes between 1.5 kb and 2 kb are also seen weakly expressed in the brain tissue, which may correspond to predicted isoforms that have different number of exons at the splice site in the CBR region. (Supplementary figure 2C). Sequencing of these minor amplicons confirmed that they are generated from variants with either exon 13 or 14 corresponding to products of about 1.7 and 1.8 kb (Supplementary figure 2D). The size and sequence of 1.7 kb product shows a match to a predicted mouse C3G isoform X8 (XM_030246488.1), that has 13th exon but lacks 14th exon^68^.

The 138 amino acids arising from exons 13 and 14 are well conserved in predicted C3G transcripts from various species (Supplementary figure 4A) suggesting that tissue specific splicing at this site may be an essential feature. Adult human brain tissue obtained during surgical resection from an epilepsy patient was examined for C3G expression using three different antibodies that detect C3G. Polypeptide of 175 kDa was seen in human brain sample similar to that seen in adult mouse brain (Fig. 1F). Two human cell lines K562, and IMR-32 showed expression of only the 140 kDa C3G isoform (Supplementary figure 1D). RNA from human brain tissue showed the presence of additional exons similar to those seen in mouse C3G, as confirmed by PCR using hu_C3G_ISO primers which showed a 2 kb amplicon (Supplementary figure 4B). Sequencing of 2 kb amplicon showed the presence of basepairs corresponding to sequences present in mouse C3G isoform 1 (Supplementary figure 4C). Various transcripts were found for human RapGEF1 in RefSeq database, derived by automated computational analysis using gene prediction method: Gnomon. One of the predicted human cDNAs, transcript variant X10, mRNA: XM_011518575.3 showed insertion of 414 bp similar to mouse isoform 1 (Supplementary figure 4D)^68^. Our results therefore confirm brain specific expression of a human C3G isoform with additional exons in the CBR region.

The expression and localization of C3G in cells of the adult mouse brain was examined by immunohistochemistry. Specific staining for C3G was predominantly seen in the cytoplasm of cortical neurons when examined using H300 or C-19 antibodies. It was observed that a subset of cells was negative for C3G staining. Similar section processed without addition of primary antibody (Blank) does not show signals specific to cells (Fig. 1G). We examined the expression of C3G in neuronal and glial cell lines derived from human or mouse, by western blotting as well as immunofluorescence. All cell lines examined, irrespective of whether they were of neuronal or glial cell origin, showed C3G polypeptides of 140 kDa, similar to that seen in a mouse myoblast cell line, C2C12 (Supplementary figure 5A). Polypeptides of 175 kDa seen in mouse and human brain tissue were not expressed in these cells. The subcellular localization of C3G was also examined in some of these cells by indirect immunofluorescence. C3G staining was seen in the nucleus as well as cytoplasm, though the relative intensities in these compartments differed in the various lines (Supplementary figure 5B). These results suggest that mouse and human brain tissue express the 175 kDa C3G protein generated by alternately spliced mRNA, but its expression is lost in neuronal and glial cell lines.

### C3G is expressed in neurons and mitral cells of the olfactory bulb

**Figure 2:**
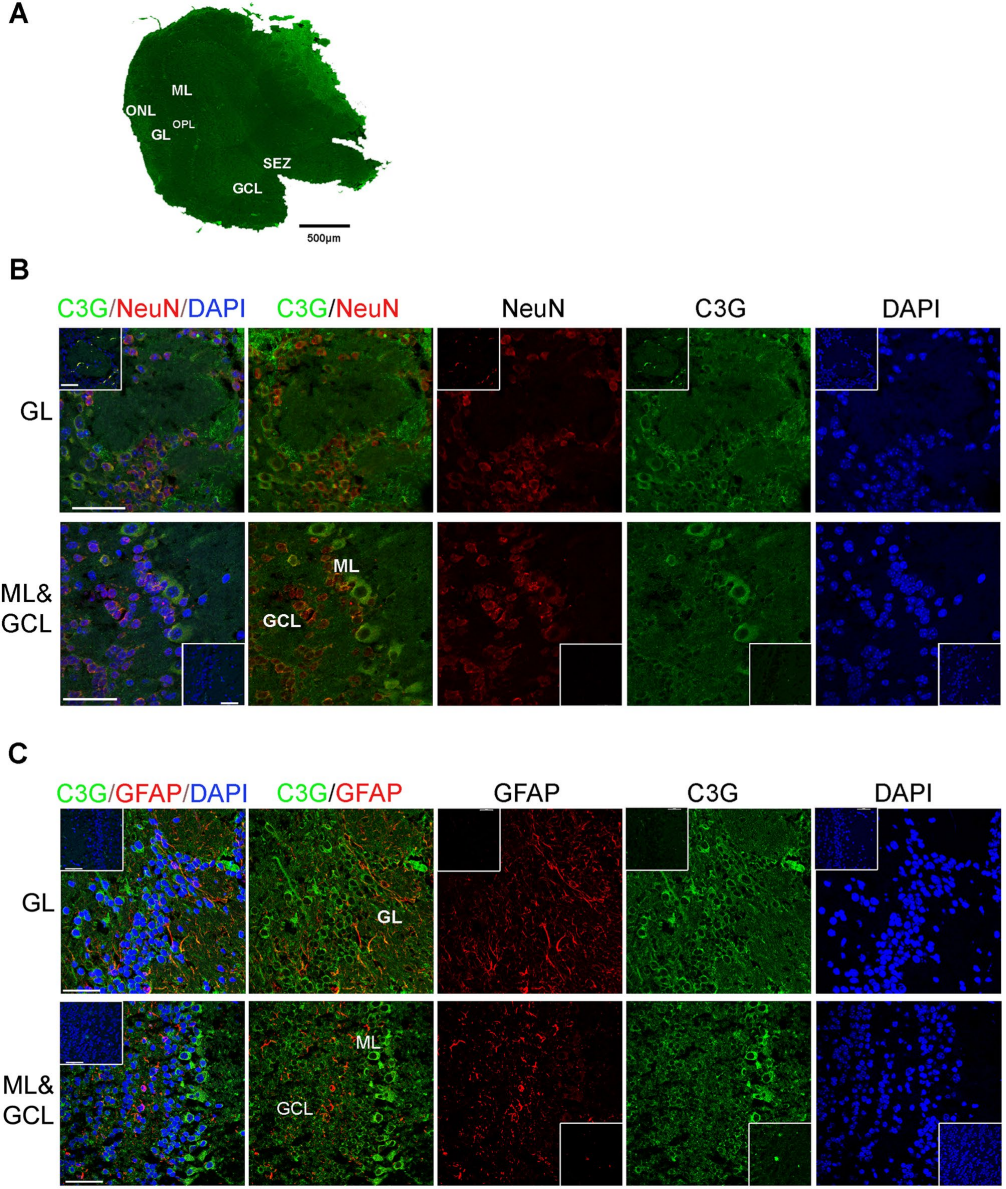
C3G expression in olfactory bulb of 3 months-old mouse brain. (**A**) Reconstructed image of olfactory bulb section stained for C3G (green), using multiple overlapping fields indicating the location of various sub-domains. Scale bar, 500 µm. (**B**,**C**) Panels show confocal microscope images of GL, ML and GCL regions of olfactory bulb tissue sections, co-stained with C3G (green) and NeuN (red) (**B**), or C3G (green) and GFAP (red) (**C**). The corresponding blanks are shown as inset images of the same magnification. Secondary antibodies anti-rabbit Alexa-488, anti-mouse Alexa-594 (NeuN) and anti-goat Alexa-546 (GFAP) were used. Scale bar, 50 µm. *ONL* olfactory nerve layer, *GL* glomerular layer, *OPL* outer plexiform layer, *ML* mitral cell layer, *GCL* granule cell layer, *SEZ* subependymal zone.

The expression of C3G was examined in transverse sections of olfactory bulbs from 3-month-old mice stained for C3G along with NeuN or Glial Fibrillary Acidic Protein (GFAP). Location of various domains in the olfactory bulb and expression of C3G is shown in the low magnification reconstructed image (Fig. 2A). Examination of sections at higher magnification showed C3G expression in all sub-regions of olfactory bulb. Neurons of the glomerular layer (GL) that were positive for NeuN, showed co-expression of C3G whereas the GFAP positive cells in this area did not show any detectable expression of C3G (Fig. 2B,C). Mitral cells in mitral cell later (ML) function as the main efferent component of the olfactory bulb by receiving odor sensing inputs and forwarding them to different parts such as piriform cortex, entorhinal cortex and amygdala, and are characterized by their large size^70,71^. These cells showed predominant expression of C3G. Tufted cells in the outer plexiform layer (OPL) also express C3G. Neurons in the granular cell layer (GCL) were positive for both NeuN and C3G. The astrocytes in the GCL did not show expression of C3G (Fig. 2C).

### Expression of C3G is high in CA3 region of hippocampus

**Figure 3:**
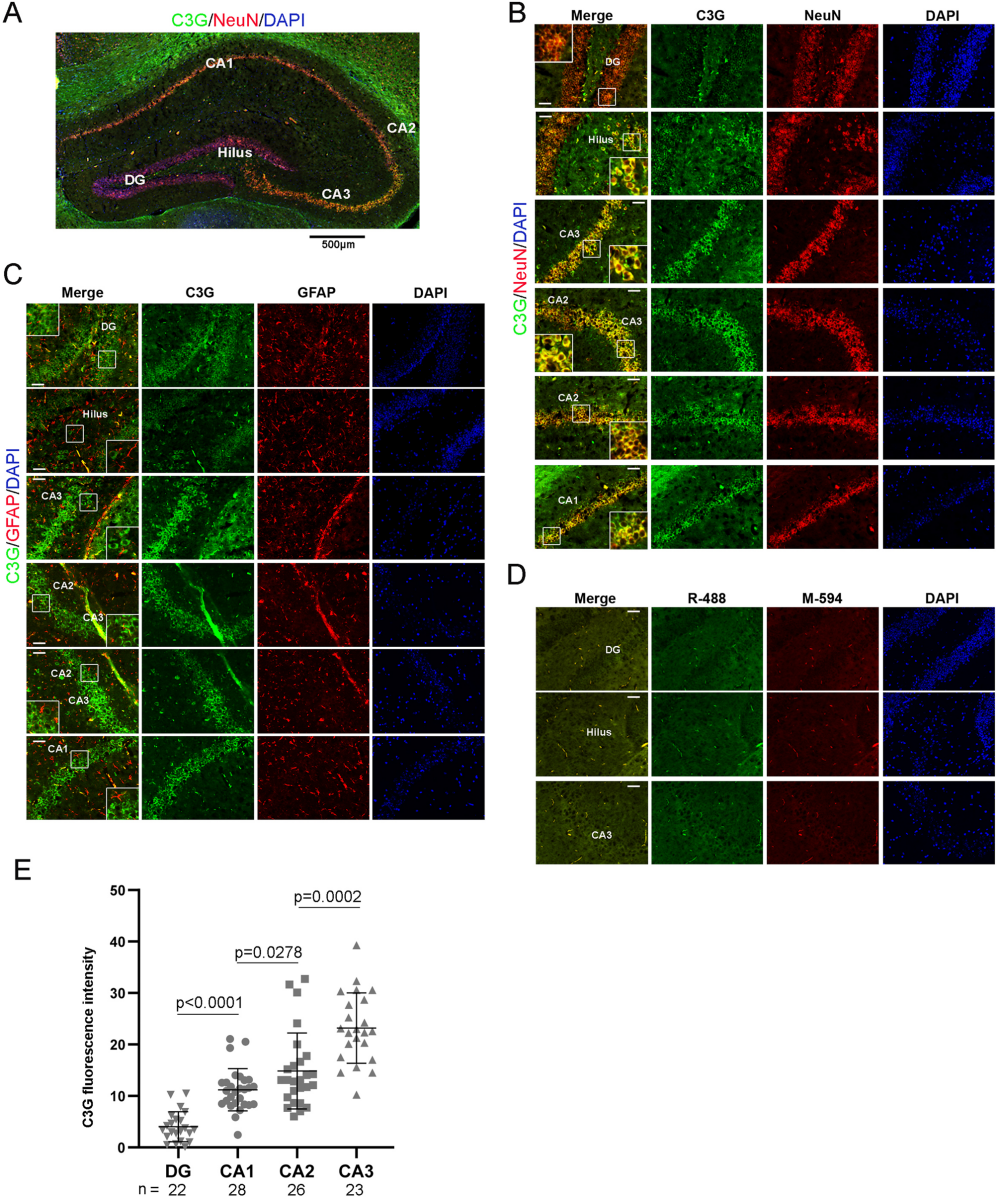
C3G expression in hippocampus of 3 months-old mouse brain. (**A**) Reconstructed image of hippocampus, stained with C3G (H300) (green) and NeuN (red). Scale bar, 500 µm. (**B**,**C**) Panels show images of different regions of hippocampus stained with C3G (H300) (green) and NeuN (red) (**B**), or C3G (H300) and GFAP (red) (**C**). Insets show enlarged images of the indicated regions. (**D**) Sections of indicated regions processed without addition of primary antibodies are shown as blanks. Secondary antibodies anti-rabbit Alexa-488 (R-488) and anti-mouse Alexa-594 (M-594) were used. (DG, Dentate gyrus; CA1, CA2 and CA3, Cornu Ammonis areas). Images were taken using Axioimager Z1 microscope. Scale bar, 50 µm. (**E**) Scatter plot shows C3G fluorescence intensities in various sub domains of the hippocampus. Horizontal line, and error bar represent mean ± standard deviation. p values are shown to indicate significance of difference in intensities at 95% confidence limit between the indicated regions.

Transverse brain sections through the hippocampal region were stained for C3G, and NeuN, or GFAP. Images taken using a 5 × objective were reconstructed by photomerging to show the architecture of the whole region (Fig. 3A). Examination using higher objective showed the expression of C3G in all neurons of the hippocampus (Fig. 3B). Expression was seen in the granular cells of dentate gyrus (DG) as well as pyramidal cells of Cornu Ammonis (CA) regions. C3G fluorescence intensity quantitation showed highest expression in CA3 region of the hippocampus (Fig. 3E). Granular cells and pyramidal cells were identified by their morphological characteristics^72^. Granular cells of DG are smaller and closely packed, having polarized morphology, with the cell body projecting into dentate molecular layer and axons projecting towards hilus and CA3 pyramidal cell layer. Pyramidal cells in the CA sub-region have a large cell body, and are seen as a densely packed layer curving into a 'U' shape. Sections processed without addition of primary antibodies are shown as blanks (Fig. 3D). Neurons of the hippocampus showed predominantly cytosolic localization. NeuN was seen in both nucleus and cytosol. C3G expression was not seen in any of the GFAP positive cells in this region (Fig. 3C).

### Expression of C3G in the cerebral cortex and other brain regions

We examined the expression of C3G in cells of different sub-regions of cerebral cortex using a transverse section from the mid brain. Supplementary figure 6A shows a reconstructed image indicating various regions. Neurons in the retrospenial (RSP) area, somatomotor, auditory and olfactory regions showed positivity for C3G, when probed with H300 antibody along with NeuN (Supplementary figure 6B). C3G was also present in NeuN negative neurons of the thalamus. Most of the neurons in the regions of the fiber tract such as corpus collosum (CC), fimbria (fi) and corticospinal tract (cpd and int) that were negative for NeuN, showed positivity for C3G. It was seen that a subset of cells in these areas, particularly the internal capsule, which were NeuN negative, did not also show C3G staining. Neurons belonging to different regions are known to serve varied functions in the mammalian brain^70,73–76^. It therefore appears that C3G is expressed in all neuronal sub-types, irrespective of their distinct functions. Expression of C3G in mouse brain cortical sections was also validated using an alternate C3G antibody, C-19 that targets residues in the C-terminal domain of C3G (Supplementary figure 7A). C3G co-expression with NeuN was seen in the cortical neurons. In other brain regions also, C3G detected using the C-19 antibody showed similar pattern of expression to that seen with H300 antibody (Supplementary figure 7B).

### Age dependent expression of C3G in hippocampus and olfactory bulb

**Figure 4:**
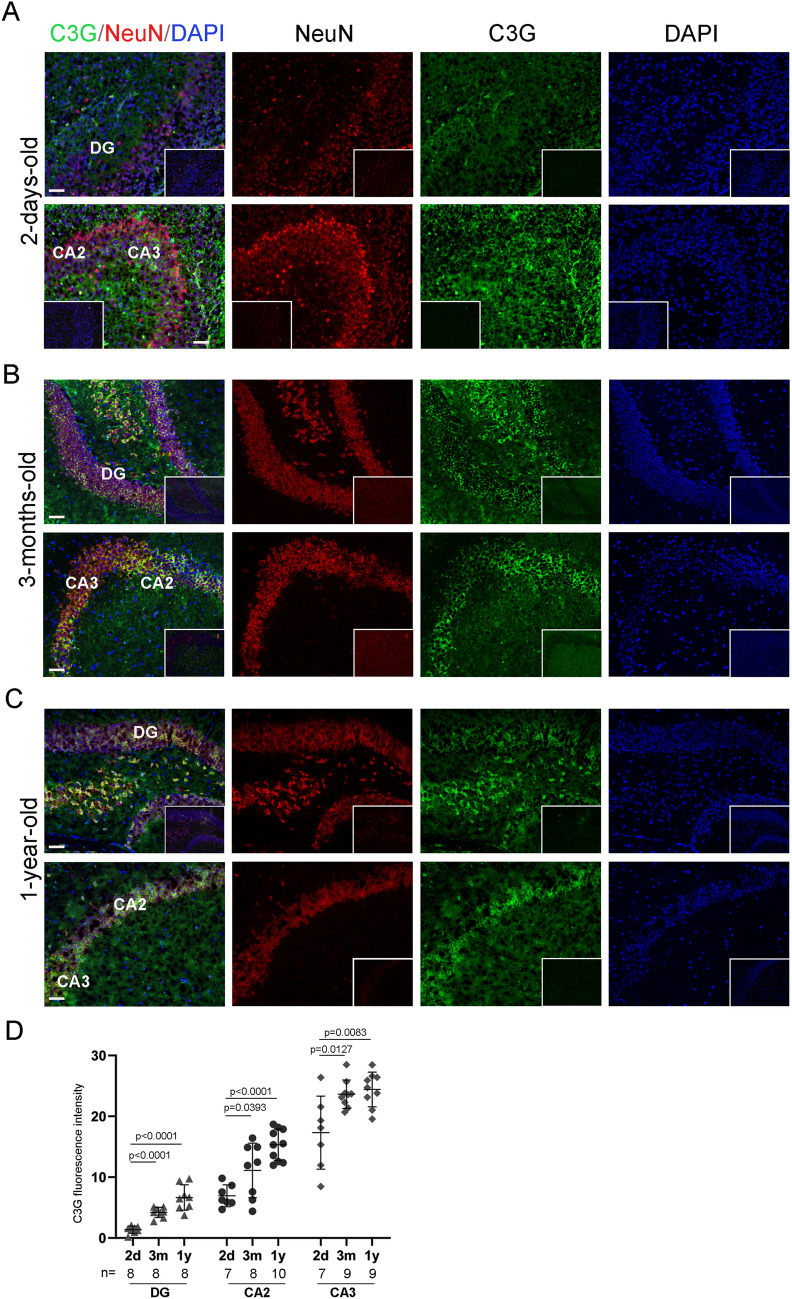
C3G expression in hippocampus of mice of different age groups. Panels show images of hippocampal sections of (**A**) 2-day-old mouse, (**B**) 3-months-old mouse and (**C**) 1-year-old mouse, stained with C3G (H300) (green) and NeuN (red). Sub-regions of hippocampus are labelled as DG, dentate gyrus; CA1, CA2 and CA3, Cornu Ammonis areas. Inset images are blanks of the same magnification. Secondary antibodies anti-rabbit Alexa-488 and anti-mouse Alexa-594 were used. Images were taken using Nikon ECLIPSE microscope. Scale bar, 50 µm. (**D**) Quantitation of C3G expression in hippocampal regions of mice of different age groups. Scatter plot shows fluorescence intensity of C3G in the indicated regions from mice belonging to different age-groups. Horizontal line, and error bar represent mean ± standard deviation. p values are shown to indicate significance of difference in intensities at 95% confidence limit between the indicated regions.

We examined the expression of C3G in hippocampal sections of brains collected from neonatal (2 days-old), adult (3-months-old) and aged (1-year-old) mice. Western blotting of whole brain lysates obtained from these age groups did not show significant difference in C3G levels (Fig. 1A). The hippocampal region is poorly differentiated in neonatal mice and showed weak expression of C3G and NeuN in the DG (Fig. 4A). Distinct expression of C3G was seen in the neurons of CA2 and CA3 regions of 3-months old mice (Fig. 4B). NeuN expression was primarily restricted to neurons present in the CA2 and CA3 regions of the neonatal brain, possibly due to differential maturation of hippocampal neurons^77,78^. Similar to brains from 3-months-old mice, 1-year-old mice showed prominent C3G expression along with NeuN in DG as well as CA2 and CA3 regions (Figs. 3B, 4C). Though whole brain lysates from mice of these age groups did not show significant differences in total C3G protein expression, quantitation of fluorescence intensity in sub-regions showed weak expression in neonatal hippocampal neurons compared to corresponding regions in 3-months-old and 1-year-old mice (Fig. 4D). Age-related differences in C3G expression between neonatal hippocampus and adult hippocampus suggests that C3G expression increases during maturation of neurons in the hippocampus.

C3G expression was also examined in the olfactory bulb of mice from different age groups. Low magnification images showed nearly uniform expression of C3G in the neonatal (1-day-old) olfactory bulb, which is poorly differentiated (Supplementary figure 8A). Examination of cells co-labelled for expression of C3G and NeuN at higher magnification showed C3G expression in all olfactory bulb cells of the neonatal brain while NeuN was weakly seen in a subset of cells (Supplementary figure 8B). Cells of the mitral layer (ML) showed high levels of C3G expression in 3-months-old as well as 1-year-old mice. Neurons of the granule cell layer (GCL) showed strong expression of NeuN and moderate expression of C3G in 3-months-old and 1-year-old mice.

### Expression of C3G in embryonic mouse brain and human cerebral organoids

**Figure 5:**
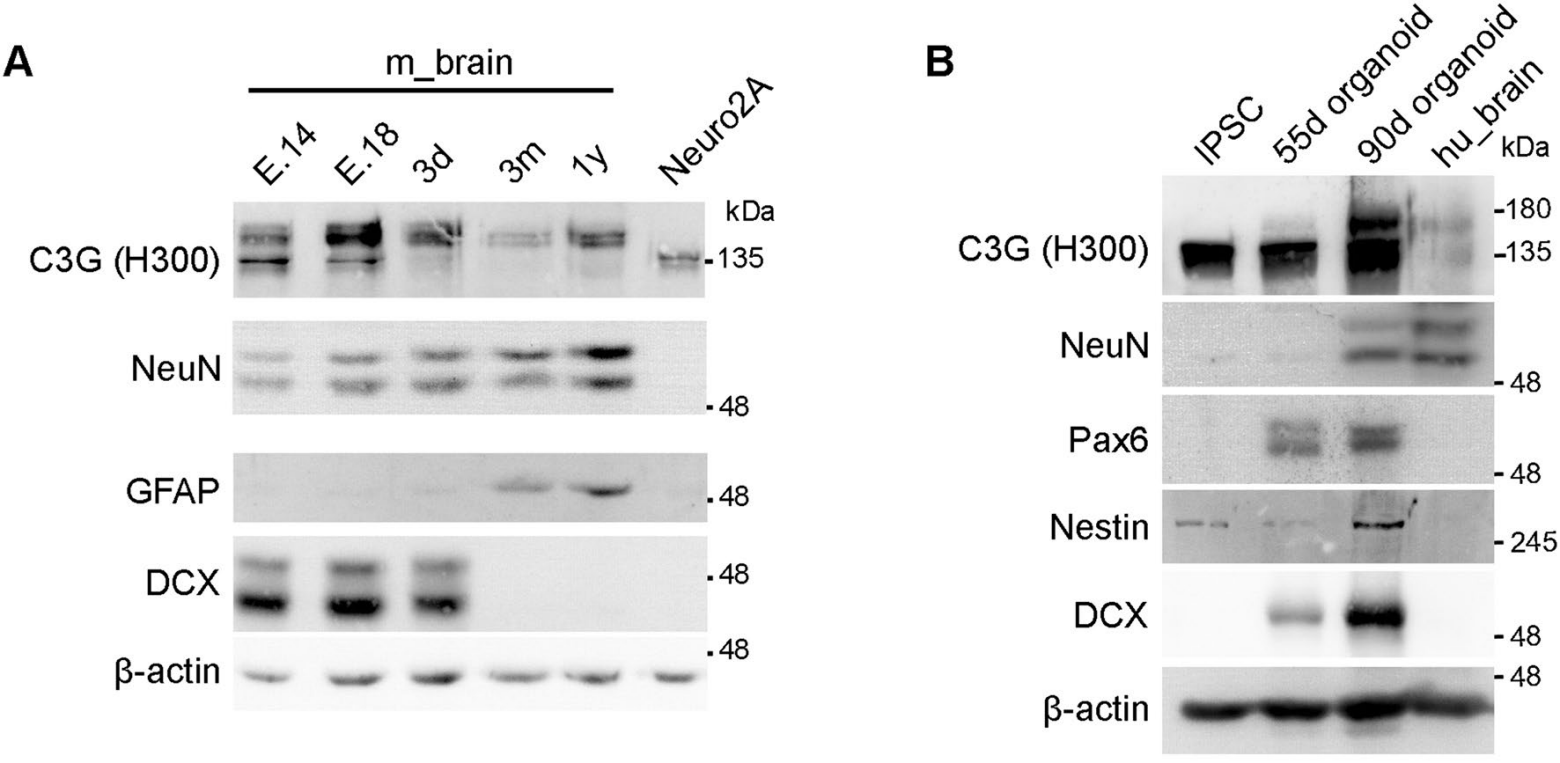
C3G expression in embryonic mouse brain and human cortical organoids. (**A**) 14th (E.14) and 18th (E.18) day mouse embryo, 3-days-old (3d), 3-months-old (3 m) and 1-year-old (1y) mouse brain lysates were subjected to western blotting by probing the same membrane for C3G (H300), NeuN, GFAP and DCX. The upper part of the membrane, cut above 75 kDa, was probed for C3G (H300 antibody). The lower part between markers 75 kDa and 25 kDa was probed for NeuN, GFAP and DCX (blots were deprobed after each antibody probing). Neuro2A cell lysate was used as positive control for expression of 140 kDa isoform of C3G (Q91ZZ2). β-Actin was used as loading control. (**B**) Expression of C3G in human cerebral organoids. Lysates prepared from iPSCs, 55-day (55d), 90-day (90d) organoids and human brain lysate were subjected to western blotting using indicated antibodies. The blots were cut and processed as indicated in (**A**).

To understand the role of C3G in mouse brain development, we examined its expression in mouse brain lysates from embryonic-day 14 and 18 (E14 and E18). Western blotting showed the presence of the 175 kDa C3G protein in brain tissue from all age groups (Fig. 5A). Unlike adult brain tissue, embryonic brain samples expressed the 140 kDa C3G polypeptide, found predominantly in cell lines and testis tissue. Similar to the differential expression of the two isoforms detected by RT-PCR (Fig. 1D), E14 brain lysate showed higher expression of the 140 kDa polypeptides relative to the 175 kDa products. At these developmental stages, neuronal precursor cell marker DCX is expressed, while mature neuron marker is weakly expressed. GFAP expression was not observed (Fig. 5A).

To examine C3G expression during human brain development, we generated cerebral organoids from iPSCs (schematic shown in Supplementary figure 9). Brain organoids grown in vitro serve as good models to recapitulate development process and organization of human brain^45^. Figure 5B shows the predominant expression of the 140 kDa C3G isoform in iPSCs as well as 55-day organoids. By 90 days of maturation, the cerebral organoids show expression of the 175 kDa isoform, concomitant with the expression of NeuN, a marker of mature neurons. Pax6, Nestin, and DCX were examined as markers of neuroectoderm, and neural precursor cells. Their expression is seen in the organoid samples, but not in adult brain tissue. These results show that expression of C3G isoforms in the human brain is developmentally regulated, with the 175 kDa isoform expressing as neurons mature.

### C3G expression in neural progenitor cells

**Figure 6:**
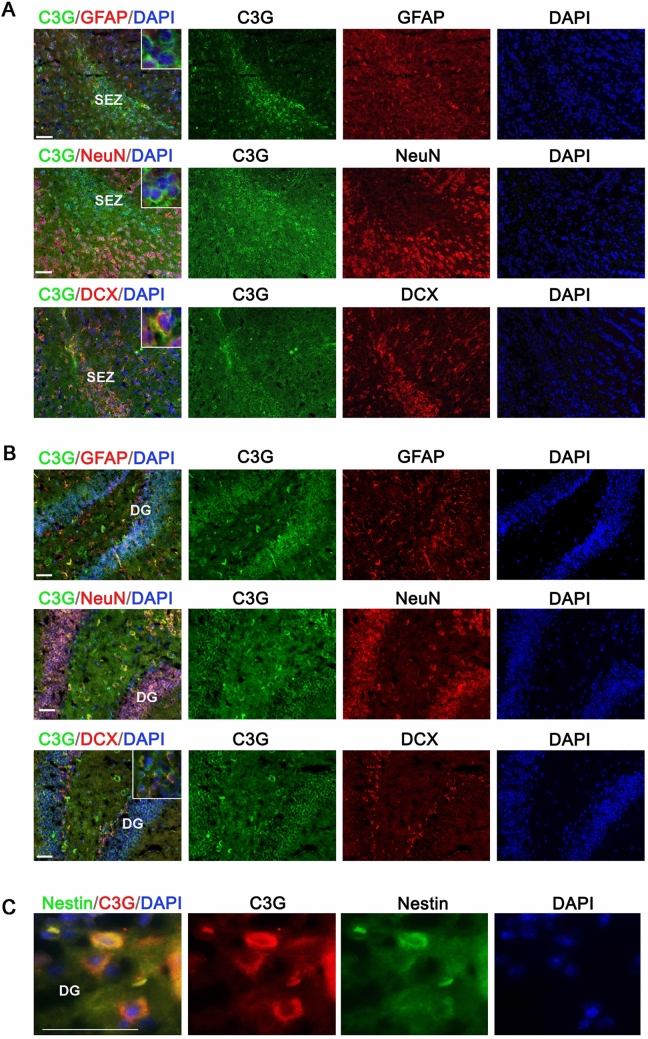
C3G expression in neural progenitor cells of olfactory bulb and hippocampus of 3-months-old mouse. (**A**) Panels show images of C3G (H300) (green)-stained SEZ (subependymal zone) region of olfactory bulb co-stained with GFAP (red), NeuN (red), or DCX (red). Inset shows enlarged images. (**B**) Panels show images of C3G (H300) (green)-stained DG (Dentate gyrus) region of hippocampus, co-stained with GFAP (red), NeuN (red), or DCX (red). Secondary antibodies anti-rabbit Alexa-488, anti-mouse Alexa-594 (NeuN) and anti-goat Alexa-546 (GFAP/DCX) were used. Insert shows enlarged images. (**C**) Panels show images of DG (Dentate gyrus) region of hippocampus, of Nestin-GFP transgenic mouse (green) co-stained with C3G (H300) (anti-rabbit Alexa-546, red). Images were taken using Axioimager Z1 microscope. Scale bar, 50 µm.

C3G is widely expressed in the developing brain collected from E14 and E18 mice as seen from transverse sections. This pattern of expression is similar to that described earlier^36^. Most of the cells at this stage show expression of C3G, and very weak expression of NeuN, as observed by immunohistochemistry (data not shown) and by western blotting (Fig. 5A). The expression of C3G in embryonic brain neurons prior to expression of NeuN suggested that C3G is expressed in NPCs. This was examined in the hippocampal and olfactory brain sections of 3-months-old mouse, by co-staining C3G with NeuN, GFAP or DCX. DCX is a microtubule associated protein expressed in adult NPCs, and used as an established marker^49^. As seen from Fig. 6A,B, C3G showed co-expression with DCX in a subset of cells in the olfactory bulb, as well as hippocampus. C3G also showed good co-localization with DCX in precursor cells of the olfactory bulb. The expression of C3G in NPCs was also confirmed by examining its expression in Nestin positive cells of brain sections obtained from a transgenic mouse expressing GFP-Nestin (Fig. 6C).

## Discussion

The spatial and temporal pattern of C3G expression in the developing and mature mammalian brain have been examined in this study. C3G polypeptides are seen in western blots as a doublet of nearly 140 kDa in a variety of cell lines when probed with commercial or in-house generated antibodies that detect the C3G protein specifically^10,20,22,28,57^. Examination of mouse and human brain tissue showed the presence of bands with slower mobility compared to the C3G polypeptides seen in adult mouse testis or other cell lines derived from neuroblastomas or gliomas. Using three antibodies that target different epitopes in C3G, we demonstrate that the predominant polypeptides expressed in the brain are larger, with a molecular weight of 175 kDa. The expression of this form in spinal cord, and brain stem (data not shown) suggested that it is specific to the CNS. Similar to the 140 kDa polypeptide, which is known to be phosphorylated resulting in mobility shift^22^, the higher molecular weight polypeptides also ran as a doublet. This is indicative of post-translational modifications of this form, or the presence of a variant with additional residues in the brain. C3G is a substrate of c-Abl, SFKs and Cdk5, kinases expressed in the CNS^14,20,79–82^. These kinases may also be phosphorylating the 175 kDa form to cause a mobility shift.

A cDNA with an insertion of 414 base pairs in the CBR has been obtained from an adult mouse brain library. Using RNA isolated from the brain, and mass spectrometry of C3G polypeptides immunoprecipitated from brain tissue, we were able to show the expression of this longer form, which gives rise to a protein of 175 kDa. Multiple sequence alignment using BLASTp and CLUSTAL Omega tools showed that the insert sequence is highly conserved among the predicted mRNAs of various species, though there are differences in the lengths of the inserts^83–86^. The splice site is the same as that shown earlier, where an insert of 153 base pairs has been observed in rat testes^15^. Splicing in this region of CBR, appears as a hotspot generating expression of tissue specific variants. Multiple isoforms of C3G are known to exist in various species, but isoform specific functions are largely unknown. An N-ter truncated variant, p87C3G is overexpressed in chronic myeloid leukemia^87^. The insertion of additional residues may confer unique properties to this isoform, required in the brain neurons, like controlling neuronal morphogenesis and structural plasticity. Interestingly, unique phosphorylation sites for Protein kinase G, casein kinase I, and insulin receptor were found among the 138 amino acids, that were absent in the 1086 isoform, when the sequences were analyzed through NetPhos3.1 (https://www.cbs.dtu.dk/services/NetPhos/)^88^. In fact, mass spectrometry data collected from the mouse brain^89^ identified phosphorylation at amino acid S675 in a peptide present within the brain specific 175 kDa form. We propose to clone this brain specific isoform to carry out further studies and understand its unique functions.

Despite major differences in the microenvironment, gene regulatory networks, developmental processes, and 3D organization are preserved in human brain organoid models^45,46,53^. Our results from embryonic mouse brain, human cortical organoids and adult mouse and human brain indicate similarity in developmental regulation of the C3G isoforms between mouse and humans. Our results also validate the utilization of brain organoids grown in culture for studying splicing that occurs during development.

C3G immunostaining was seen only in neurons and not detected in GFAP positive cells. Though we cannot rule out the possibility of very low expression below detection limits in astrocytes, our findings are consistent with data obtained by proteomic analysis of different cell types in the mouse brain^90^. Many other proteins like Serine racemase, Eps8, and HRD1 are known to be expressed only in neurons^91–93^. During development of the nervous system, neurogenesis precedes gliogenesis^94^. It is known that inducers of neuronal fate, like, Neurogenin, suppress glial differentiation^95^. As a promoter of neuronal differentiation, C3G may be functioning as a suppressor of glial cell differentiation, and its expression is shut off in glial cells of the adult mammalian brain. C3G is predominantly expressed in pyramidal neurons in the cerebral cortex and hippocampal CA regions. Its presence in granule cells of the olfactory bulb as well as hippocampus suggested that it is expressed in both GABAergic as well as glutamatergic neurons. In neonatal olfactory bulb, all cells show C3G expression irrespective of their differentiation. Adult brain neurogenic areas, the subependymal zone of the olfactory bulb and the dentate gyrus of the hippocampus, which are positive for DCX and Nestin, also showed expression of C3G indicating that its expression is not restricted to differentiated neurons. But, identification of the isoforms expressed in these cells will require generation of antibodies that specifically recognize the 175 kDa isoform. Embryonic brain, largely composed of undifferentiated cells, and lacking expression of either NeuN or GFAP showed the presence of C3G, suggesting its possible function in proliferating precursor cells as well as differentiated neurons.

The 175 kDa protein predominantly seen in western blots of brain tissue is the form primarily expressed in neurons of the adult brain, which are in a non-proliferating state. In contrast, the 140 kDa protein is the only form detected in neuronal as well as glial cell lines derived from tumors. Many miRNAs show altered expression in gliomas^96,97^, and it is known that C3G is one of the targets of miR-124^98^. Therefore, repression of miR-124 could explain the increased expression of C3G seen in glioma cell lines. We also observed that the cell lines derived from tumors show the presence of significant amounts of C3G in the nucleus, unlike normal brain neurons and NPCs which show predominant cytoplasmic staining. We have earlier observed that compared to cells derived from other tissues, IMR-32 cells, derived from a neuroblastoma show significantly higher levels of nuclear C3G^22^. It therefore appears that external cues and alterations leading to tumorigenicity regulate expression and localization of C3G. It is possible that immortalized neuronal cell lines capable of continued proliferation, lose expression of the 175 kDa isoform and gain expression of the 140 kDa C3G isoform. The 175 kDa isoform seen in adult brain, may be inhibitory to cell proliferation.

Since C3G plays an important role in neuronal differentiation, and mice with C3G deficiency show defects in neuronal maturation and migration, it was not surprising to see that C3G is expressed in embryonic brain and also in adult NPCs. C3G is required for multipolar to bipolar transitioning in developing neurons^36^, and the activity of C3G targets like Rap1, regulate synaptic function, spine morphology, and stability^99–102^. Neurons in the CA3 region of the hippocampus show a higher degree of connectivity and play specific role in memory^103^. The higher expression of C3G in this region is suggestive of an important role for C3G in maintaining structural and functional properties of these neurons. During development, the program regulating division and self-renewal, or exit from the cell cycle and differentiation is tightly controlled, and imbalances can lead to severe functional consequences. This is evident from the phenotype of mice lacking normal levels of C3G.

## Methods

### Cell lines and organoid generation

Neuro2A (Cat no: CCL-131, RRID:CVCL_0470), RGC-5 (Cat no: PTA-6600, RRID:CVCL_4059), IMR-32 (Cat no: CCL-127, RRID:CVCL_0346), HN25, NSC-34, U-87 MG (Cat no: 300367/p658_U-87_MG, RRID:CVCL_0022), LN-229 (Cat no: CRL-2611, RRID:CVCL_0393), HNGC-2, K562 (Cat no: CCL-243, RRID:CVCL_0004), and 661W cell lines (provided by Dr. Muyyad R. Al Ubaidi) were maintained in Dulbecco’s modified Eagle Medium (DMEM) supplemented with 10% fetal bovine serum (FBS) at 37 °C in a CO_2_ incubator. DMEM supplemented with 20% FBS was used to maintain C2C12 cells. All experiments were carried out with exponentially growing cells. Human induced pluripotent stem cells (hiPSC) F2-3F1 used for generation of cerebral organoids have been described^104^. IEC sanction no. IEC-71/2019, was obtained for work on human iPSCs. They were maintained in E8 medium and were differentiated into cerebral organoids using the protocol described earlier^105^. The organoids were allowed to mature for 3 months. 55-day and 90-day organoids were used for examining C3G protein.

### Materials

Anti-rabbit C3G (H300) [Cat no: sc-15359; RRID:AB_2177452] and C3G (C-19) [Cat no: sc-869; RRID:AB_2177454], anti-mouse C3G (G4), anti-goat Doublecortin (DCX)(C-18) [Cat no: sc-8066; RRID:AB_2088494], anti-goat Nestin (G-20) [Cat no: sc-21248; RRID:AB_2148925] and normal mouse IgG [Cat no: sc-2025; RRID:AB_737182] were purchased from Santa Cruz Biotechnology. Anti-mouse NeuN [1B7] (Cat no: ab104224), anti-goat GFAP [Cat no: ab53554; RRID:AB_880202] and anti-mouse Pax6 (AD2.38) [Cat no: ab78545; RRID:AB_1566562] were procured from Abcam. Anti-mouse β-actin [Cat no: MAB1501; RRID:AB_2223041] was from Millipore. All fluorescent labelled secondary antibodies were obtained from Thermo Fisher Scientific. C3G 3F6mAb, mouse monoclonal antibody, was raised against recombinant CBR in the lab, and characterized^57^. C3G-GFP plasmid has been described earlier^12^.

Protein molecular weight marker prism ultra was purchased from Abcam. DNA ladder, Gene Ruler (100 bp–5 kb) was obtained from Thermo Fisher Scientific. 1 kb ladder was obtained from NEB. Protein A/G agarose beads (Cat no: sc-2003) were purchased from Santa Cruz Biotechnology. Protease inhibitor cocktail (Cat no: 11873580001) was obtained from Roche. Trypsin used for in-gel digestion was from Roche. Acetonitrile and formic acid were from Biosolve (Mass spectrometry grade).

### Animals and tissue processing

The work was carried out after approval by the Institutional animal ethics committee (IAEC) of CCMB, no. IAEC 08/1017, and the protocols strictly follow the guidelines of Committee for the Purpose of Control and Supervision of Experiments on Animals (CPCSEA), Government of India for animal welfare. Institutional ethics committee (IEC) of CCMB approval no. IEC 57/2017 was obtained for procuring human brain tissue. Experiments were undertaken after obtaining the informed and written consent of each subject. None of them were below 18 years of age. The study conforms with The Code of Ethics of the World Medical Association.

Wild-type C57 black mice (RRID: IMSR_JAX:000664) and Nestin-GFP transgenic mice, expressing GFP, under the control of nestin gene regulatory regions, were used in this study^106^. Animals were deeply anesthetized with ether and cardiac perfusion performed with normal saline followed by 4% paraformaldehyde (PFA) in PBS. Brains were collected after decapitation, fixed in 4% PFA overnight at 4 °C and transferred to 30% sucrose solution until they settled down. For cryosections, fixed brains were sliced from the mouse brain matrix and mounted in OCT (Optimal cutting temperature) freezing medium, and 10 µm transverse sections taken using a Leica CM1950 cryostat. Sections were then collected on slides and used for immunohistochemistry. All results were validated by examining 3 or more sections from each brain region, obtained from 3–5 animals of each age group. Western blotting and transcript analysis was carried out using tissues prepared from three animals of each group.

### Indirect immunofluorescence and microscopy

Immunohistochemistry was carried out using tissue sections on slides. Slides were rehydrated by sequential immersion in gradient alcohol (100%, 90% and 70%), for 5 min each, followed by a 5 min wash in distilled water. Antigen retrieval was done by immersing the slides in trisodium citrate buffer (pH 6) for 24 min, in a boiling water bath, and distilled water wash for 5 min at room temperature. Sections were then blocked using 2% BSA for 1 h at room temperature followed by primary antibody incubation for 2 h at room temperature or overnight at 4 °C. The slides were washed and incubated with the secondary antibody for 1 h at RT. After washes, the slides were mounted with glycerol containing anti-fade and DAPI. Adjoining tissue sections, in all cases were processed similarly, but without addition of primary antibody, to serve as blanks for secondary antibodies.

Dual labelling was carried out by sequential incubations of the two primary and corresponding secondary antibodies. Anti-rabbit Alexa-488 (green) secondary antibody was used for C3G staining, except in case of Nestin-GFP mouse brain section (Fig. 6C), where anti- rabbit Alexa 546 secondary antibody was used for C3G staining. H300 antibody was used to detect C3G in all experiments, unless otherwise mentioned. Anti-mouse Alexa-594 (red) secondary antibody was used for NeuN staining. Anti-goat Alexa-546 (red) secondary antibody was used for GFAP and DCX staining. For examining endogenous C3G in various murine and human-origin cell lines, cells were cultured on 18 mm coverslips, fixed with cold methanol and indirect immunofluorescence carried out as described earlier^64^.

### Image analysis and statistics

Images were captured using Nikon ECLIPSE microscope, Leica TCS SP8 Confocal microscope (Leica Microsystems, Germany) or Axioimager Z1, Carl Zeiss (Germany). Images from the Confocal Z stacks were merged and processed using the Leica Application Suite. Constant image acquisition parameters were used for capturing images of all samples from a given experiment. Image reconstruction was carried out by capturing multiple images of various mouse brain sections using a 5 × objective of Axioimager Z1 fluorescence microscope (Carl Zeiss). Complete image was reconstructed using Photomerge tool in Adobe Photoshop CS3 software. Fluorescence intensity quantitation was carried out using ImageJ software. Several 25 μm^2^ areas in the DG and CA regions from multiple fields/sections of the hippocampus were marked for C3G fluorescence intensity quantitation, and adjusted with average background intensity in areas of the same section lacking cells. Data obtained from multiple sections of indicated brain regions belonging to 3 animals were averaged to assess differential expression of C3G. We used unpaired t-tests between pair of regions to determine the statistical significance of the intensities between the regions. The p-values at 95% confidence interval and number of samples (n) are shown in the figures.

### Western blotting

Whole cell lysates were prepared from cells in Laemmli buffer, western blotting carried out as described earlier^20^. Brain and testis lysates were prepared by homogenizing in modified RIPA buffer (50 mM Tris–HCl, pH 7.4; 150 mM NaCl; 1 mM EDTA; 1% NP-40; 0.5% sodium deoxycholate, 0.1% SDS and 1× Protease inhibitor cocktail). Subsequently, samples were boiled in 1× Laemmli buffer and subjected to SDS-PAGE, as described earlier^20^. 20 µg of total protein was loaded in each lane after quantitation^107^. After transfer, the membranes were cut based on size of expected bands of various antigens, and probed simultaneously, whenever commercial and previously characterized primary antibodies were used. The images of the membranes (some full length, and some cut to size to save primary antibodies/avoid deprobing multiple times) used are shown in the supplementary information provided with original scans. Deprobing was carried out when the same part of the membrane was used to probe for more than one antigen. Bands were detected using Vilber-Lourmat Chemiluminescence system (Germany). Western blot panels shown in each figure are those obtained from the same gel.

### Semi-quantitative RT-PCR and sequencing

Total RNA was prepared from mouse tissues, adult human brain and C2C12 cell line using Trizol (Invitrogen) reagent. RNA was converted to cDNA using reverse transcriptase (Invitrogen SuperScript III First-Strand Synthesis System kit). PCR was carried out using the prepared cDNA for amplification of C3G isoforms and β-actin. Mouse specific primers m_C3G_ISO-F (5′-GAACAAGCACATGCTGGCCTA-3′) and m_C3G_ISO-R (5′-CCTAGGCTTGATCTTCAGAGAG-3′) for distinguishing isoforms with and without the additional exons, were used to amplify C3G from cDNA of mouse tissues and C2C12. Actin-F (5′-GGCTGTATTCCCCTCCATCG-3′) and Actin-R (5′-CCAGTTGGTAACAATGCCATGT-3′) were used to amplify actin, used as an internal control. PCR was performed in Eppendorf Master Cycler with, Phusion High-Fidelity DNA Polymerase (Thermo Fisher Scientific). The following PCR conditions were used for amplification of C3G: initial denaturation was at 98 °C for 30 s, followed by 30 cycles of denaturation at 98 °C for 10 s; annealing at 65 °C for 30 s, and extension at 72 °C for 2 min. Final extension was for 5 min at 72 °C. Amplified PCR products were examined and pictures captured using Vilber-Lourmat Gel Documentation system (Germany). The PCR fragments were eluted and sequenced to confirm that the amplicons are generated from alternate splice variants. Mouse specific m_N-term_F primer (5′-CGTTCTCATCTCTCCTCCTTC-3′) and m_N-term_R primers (5′-ACAGTTGTCACCATCTCCTTATC-3′) were used to determine presence or absence of exon 4 in mouse brain cDNA.PCR conditions used are similar as above, except annealing temperature used was 48 °C. Human specific primers hu_C3G_ISO-F (5′-CAAACACATGCTGGCCTACAT-3′) and hu_C3G_ISO-R (5′-CTGGGTTTAATTTTCAGAGACAG-3′) were used to amplify C3G from human brain cDNA, in order to distinguishing isoforms with and without additional exons. PCR was performed as mentioned above. PCR conditions used was: initial denaturation was at 98 °C for 30 s, followed by 30 cycles of denaturation at 98 °C for 10 s; annealing at 48 °C for 30 s, and extension at 72 °C for 3 min. Final extension was for 10 min at 72 °C.

### Immunoprecipitation and mass spectrometry (LC–ESI–MS/MS)

3-months-old mouse whole brain lysate was prepared by homogenizing in modified RIPA buffer (50 mM Tris–HCl, pH 7.4; 150 mM NaCl; 1 mM EDTA; 1% NP-40; 0.5% sodium deoxycholate, 0.1% SDS and 1× protease inhibitor cocktail). 50 µg of protein was diluted 30 times in a microfuge tube with immunoprecipitation wash buffer (25 mM Tris–HCl, pH 7.4; 150 mM NaCl; 5 mM EDTA and 1× protease inhibitor cocktail). 5 µg of C3G (G4) antibody was added to the tubes and rotated overnight at 4 °C (brain lysate incubated with mouse IgG antibody was used as control for IP). 20 µl of Protein-A/G agarose beads were then added to the tubes and rotated for one hour at 4 °C. The beads were washed; immunoprecipitated proteins were denatured using Laemmli buffer and boiled for 5 min. Proteins were resolved on NuPAGE 4%–12% Bis–Tris Protein Gel (Invitrogen) using MES buffer (100 mM MES, 100 mM Tris–HCl, 2 mM EDTA, 7 mM SDS) at 200 V for 30 min, fixed and stained with Coomassie brilliant blue. Preparation of gel slices, reduction, alkylation, and in-gel protein digestion was carried out for the 175 kDa band as described by Shevchenko et al. 1996^108^. Subsequently, peptides were desalted and enriched according to Rappsilber et al. 2003^109^.

Peptides eluted from desalting tips were dissolved in 2% formic acid and sonicated for 5 min. Peptide fractions were analyzed on Q Exactive (Thermo Fisher Scientific) interfaced with nanoflow LC system (Easy nLC 1200, Thermo Fisher Scientific). Then they were separated on a PepMap RSLC C18 pico-Frit nanocapillary column (75 μm × 15 cm; 3 μm), using a 60-min linear gradient of the mobile phase [5% ACN containing 0.1% formic acid (buffer-A) and 90% ACN containing 0.1% formic acid (buffer-B)] at a flow rate of 300 nL/min. Full-scan MS spectra (from m/z 400 to 1750) were acquired followed by MS/MS scans of top 10 peptides with charge states 2 to 6.

For peptide identification, raw MS data files were loaded onto computational platform Proteome Discoverer (Ver.1.4.1.12) and processed using search algorithm- SEQUEST HT, against UniProt Unreviewed *Mus musculus* (release 2019.04 with 2,41,774 entries), Reviewed *Mus musculus* (release 2019.04 with 36,089 entries) databases and a database of known contaminants. The search parameters applied in the database searches were: enzyme specificity: *trypsin*; maximum missed cleavages: *2*; *carboxymethyl* (C) as a static modification; *oxidation* (M) as dynamic modifications; a precursor mass tolerance of *5 ppm*; and a fragment mass tolerance of *0.02 Da*. Other parameters included- minimum peptide identification-*6*, Peptide filters: Peptide confidence set at [*high*], Protein filters: minimum number of Peptides per protein -*6*; [*count peptide only in top scored proteins*] and [*count only rank 1 peptide*] were selected.

## Supplementary information

Supplementary Figures.

## Abbreviations

GEF: Guanine nucleotide exchange factor
CBR: Crk-binding region
GFAP: Glial fibrillary acidic protein
NGF: Nerve growth factor
DCX: Doublecortin
NPC: Neural progenitor cells
DG: Dentate gyrus
CA: Cornu Ammonis
RT-PCR: Reverse-transcriptase polymerase chain reaction
iPSCs: Induced pluripotent stem cells
RRID: Research Resource Identifier

## References

[1] Quilliam LA, Rebhun JF, Castro AF (2002). A growing family of guanine nucleotide exchange factors is responsible for activation of Ras-family GTPases. Prog. Nucleic Acid Res. Mol. Biol..

[2] Radha V, Mitra A, Dayma K, Sasikumar K (2011). Signalling to actin: role of C3G, a multitasking guanine-nucleotide-exchange factor. Biosci. Rep..

[3] Chiang SH (2001). Insulin-stimulated GLUT4 translocation requires the CAP-dependent activation of TC10. Nature.

[4] Gotoh T (1995). Identification of Rap1 as a target for the Crk Sh3 domain-binding guanine nucleotide-releasing factor C3g. Mol. Cell. Biol..

[5] Gotoh T (1997). Activation of R-Ras by Ras-guanine nucleotide-releasing factor. J. Biol. Chem..

[6] van den Berghe N, Cool RH, Horn G, Wittinghofer A (1997). Biochemical characterization of C3G: an exchange factor that discriminates between Rap1 and Rap2 and is not inhibited by Rap 1A(S17N). Oncogene.

[7] Kirsch KH, Georgescu MM, Hanafusa H (1998). Direct binding of p130(Cas) to the guanine nucleotide exchange factor C3G. J. Biol. Chem..

[8] Knudsen BS, Feller SM, Hanafusa H (1994). Four proline-rich sequences of the guanine-nucleotide exchange factor C3G bind with unique specificity to the first Src homology 3 domain of Crk. J. Biol. Chem..

[9] Mitra A, Radha V (2010). F-actin-binding domain of c-Abl regulates localized phosphorylation of C3G: role of C3G in c-Abl-mediated cell death. Oncogene.

[10] Shivakrupa R, Radha V, Sudhakar C, Swarup G (2003). Physical and functional interaction between Hck tyrosine kinase and guanine nucleotide exchange factor C3G results in apoptosis, which is independent of C3G catalytic domain. J. Biol. Chem..

[11] Tanaka S (1994). C3G, a guanine nucleotide-releasing protein expressed ubiquitously, binds to the Src homology 3 domains of CRK and GRB2/ASH proteins. Proc. Natl. Acad. Sci. U.S.A..

[12] Dayma K, Ramadhas A, Sasikumar K, Radha V (2012). Reciprocal negative regulation between the guanine nucleotide exchange factor C3G and beta-catenin. Genes Cancer.

[13] Kloog Y, Mor A (2014). Cytotoxic-T-lymphocyte antigen 4 receptor signaling for lymphocyte adhesion is mediated by C3G and Rap1. Mol. Cell. Biol..

[14] Mitra A, Kalayarasan S, Gupta V, Radha V (2011). TC-PTP dephosphorylates the guanine nucleotide exchange factor C3G (RapGEF1) and negatively regulates differentiation of human neuroblastoma cells. PLoS ONE.

[15] Singh SR, Swarup G (1999). Identification of a novel splice variant of C3G which shows tissue-specific expression. DNA Cell Biol..

[16] Zhai B, Huo H, Liao K (2001). C3G, a guanine nucleotide exchange factor bound to adapter molecule c-Crk, has two alternative splicing forms. Biochem. Biophys. Res. Commun..

[17] Cheerathodi M, Vincent JJ, Ballif BA (2015). Quantitative comparison of CrkL-SH3 binding proteins from embryonic murine brain and liver: implications for developmental signaling and the quantification of protein species variants in bottom-up proteomics. J. Proteomics.

[18] Ichiba T (1999). Activation of C3G guanine nucleotide exchange factor for Rap1 by phosphorylation of tyrosine 504. J. Biol. Chem..

[19] Ichiba T (1997). Enhancement of guanine-nucleotide exchange activity of C3G for Rap1 by the expression of Crk, CrkL, and Grb2. J. Biol. Chem..

[20] Radha V, Rajanna A, Swarup G (2004). Phosphorylated guanine nucleotide exchange factor C3G, induced by pervanadate and Src family kinases localizes to the Golgi and subcortical actin cytoskeleton. BMC Cell Biol..

[21] Sakkab D (2000). Signaling of hepatocyte growth factor/scatter factor (HGF) to the small GTPase Rap1 via the large docking protein Gab1 and the adapter protein CRKL. J. Biol. Chem..

[22] Shakyawar DK, Dayma K, Ramadhas A, Varalakshmi C, Radha V (2017). C3G shows regulated nucleocytoplasmic exchange and represses histone modifications associated with euchromatin. Mol. Biol. Cell.

[23] Guerrero C (1998). Transformation suppressor activity of C3G is independent of its CDC25-homology domain. Oncogene.

[24] Gutierrez-Uzquiza A (2010). C3G down-regulates p38 MAPK activity in response to stress by Rap-1 independent mechanisms: involvement in cell death. Cell Signal..

[25] Hogan C (2004). Rap1 regulates the formation of E-cadherin-based cell-cell contacts. Mol. Cell. Biol..

[26] Martin-Encabo S, Santos E, Guerrero C (2007). C3G mediated suppression of malignant transformation involves activation of PP2A phosphatases at the subcortical actin cytoskeleton. Exp. Cell Res..

[27] Radha V, Rajanna A, Mitra A, Rangaraj N, Swarup G (2007). C3G is required for c-Abl-induced filopodia and its overexpression promotes filopodia formation. Exp. Cell Res..

[28] Kumar KS (1853). C3G (RapGEF1), a regulator of actin dynamics promotes survival and myogenic differentiation of mouse mesenchymal cells. BBA-Mol. Cell. Res..

[29] Radha V, Rajanna A, Gupta RK, Dayma K, Raman T (2008). The guanine nucleotide exchange factor, C3G regulates differentiation and survival of human neuroblastoma cells. J. Neurochem..

[30] Ortiz-Rivero S (2018). C3G, through its GEF activity, induces megakaryocytic differentiation and proplatelet formation. Cell. Commun. Signal..

[31] Wu C, Lai CF, Mobley WC (2001). Nerve growth factor activates persistent Rap1 signaling in endosomes. J. Neurosci..

[32] Ohba Y (2001). Requirement for C3G-dependent Rap1 activation for cell adhesion and embryogenesis. EMBO J..

[33] Voss AK, Krebs DL, Thomas T (2006). C3G regulates the size of the cerebral cortex neural precursor population. EMBO J..

[34] Voss AK (2008). C3G regulates cortical neuron migration, preplate splitting and radial glial cell attachment. Development.

[35] Yip YP, Thomas T, Voss AK, Yip JW (2012). Migration of sympathetic preganglionic neurons in the spinal cord of a C3G-deficient mouse suggests that C3G acts in the reelin signaling pathway. J. Comp. Neurol..

[36] Shah B (2016). C3G/Rapgef1 is required in multipolar neurons for the transition to a bipolar morphology during cortical development. PLoS ONE.

[37] Ballif BA (2004). Activation of a Dab1/CrkL/C3G/Rap1 pathway in Reelin-stimulated neurons. Curr. Biol..

[38] Hirota Y, Nakajima K (2017). Control of neuronal migration and aggregation by reelin signaling in the developing cerebral cortex. Front. Cell Dev. Biol..

[39] Sekine K (2012). Reelin controls neuronal positioning by promoting cell-matrix adhesion via inside-out activation of integrin alpha5beta1. Neuron.

[40] Voss AK, Gruss P, Thomas T (2003). The guanine nucleotide exchange factor C3G is necessary for the formation of focal adhesions and vascular maturation. Development.

[41] Amiri A (2018). Transcriptome and epigenome landscape of human cortical development modeled in organoids. Science.

[42] Andrews MG, Nowakowski TJ (2019). Human brain development through the lens of cerebral organoid models. Brain Res..

[43] Camp JG (2015). Human cerebral organoids recapitulate gene expression programs of fetal neocortex development. Proc. Natl. Acad. Sci. U.S.A..

[44] Karzbrun E, Reiner O (2019). Brain organoids—a bottom-up approach for studying human neurodevelopment. Bioengineering (Basel).

[45] Lancaster MA (2013). Cerebral organoids model human brain development and microcephaly. Nature.

[46] Luo C (2016). Cerebral organoids recapitulate epigenomic signatures of the human fetal brain. Cell Rep..

[47] Qian X, Song H, Ming GL (2019). Brain organoids: advances, applications and challenges. Development.

[48] Lendahl U, Zimmerman LB, McKay RDG (1990). CNS stem cells express a new class of intermediate filament protein. Cell.

[49] Gleeson JG, Lin PT, Flanagan LA, Walsh CA (1999). Doublecortin is a microtubule-associated protein and is expressed widely by migrating neurons. Neuron.

[50] Furlanis E, Scheiffele P (2018). Regulation of neuronal differentiation, function, and plasticity by alternative splicing. Annu. Rev. Cell Dev. Biol..

[51] Grabowski P (2011). Alternative splicing takes shape during neuronal development. Curr. Opin. Genet. Dev..

[52] Mazin P (2013). Widespread splicing changes in human brain development and aging. Mol. Syst. Biol..

[53] van de Leemput J (2014). CORTECON: a temporal transcriptome analysis of in vitro human cerebral cortex development from human embryonic stem cells. Neuron.

[54] Menon S, Gupton SL (2016). Building blocks of functioning brain: cytoskeletal dynamics in neuronal development. Int. Rev. Cell Mol. Biol..

[55] Su CH, Dhananajaya D, Tarn WY (2018). Alternative splicing in neurogenesis and brain development. Front. Mol. Biosci..

[56] Dayma K, Radha V (2011). Cytoskeletal remodeling by C3G to induce neurite-like extensions and inhibit motility in highly invasive breast carcinoma cells. Biochim. Biophys. Acta.

[57] Begum Z, Varalakshmi C, Sriram D, Radha V (2018). Development and characterization of a novel monoclonal antibody that recognizes an epitope in the central protein interaction domain of RapGEF1 (C3G). Mol. Biol. Rep..

[58] Guerrero C, Martin-Encabo S, Fernandez-Medarde A, Santos E (2004). C3G-mediated suppression of oncogene-induced focus formation in fibroblasts involves inhibition of ERK activation, cyclin A expression and alterations of anchorage-independent growth. Oncogene.

[59] Hirata T (2004). Amplification, up-regulation and over-expression of C3G (CRK SH3 domain-binding guanine nucleotide-releasing factor) in non-small cell lung cancers. J. Hum. Genet..

[60] Linghu H (2006). Involvement of adaptor protein Crk in malignant feature of human ovarian cancer cell line MCAS. Oncogene.

[61] Maia V (2013). C3G forms complexes with Bcr-Abl and p38alpha MAPK at the focal adhesions in chronic myeloid leukemia cells: implication in the regulation of leukemic cell adhesion. Cell Commun. Signal..

[62] Priego N (2016). C3G knock-down enhances migration and invasion by increasing Rap1-mediated p38alpha activation, while it impairs tumor growth through p38alpha-independent mechanisms. Oncotarget.

[63] Schonherr C, Yang HL, Vigny M, Palmer RH, Hallberg B (2010). Anaplastic lymphoma kinase activates the small GTPase Rap1 via the Rap1-specific GEF C3G in both neuroblastoma and PC12 cells. Oncogene.

[64] Shakyawar DK, Muralikrishna B, Radha V (2018). C3G dynamically associates with nuclear speckles and regulates mRNA splicing. Mol. Biol. Cell.

[65] Wang Z (2006). Rap1-mediated activation of extracellular signal-regulated kinases by cyclic AMP is dependent on the mode of Rap1 activation. Mol. Cell. Biol..

[66] Yang D (2019). Silencing of C3G increases cardiomyocyte survival inhibition and apoptosis via regulation of p-ERK1/2 and Bax. Clin. Exp. Pharmacol. Physiol..

[67] AK147468.1. Mus musculus adult male brain UNDEFINED_CELL_LINE cDNA, RIKEN full-length enriched library, clone:M5C1048M04 product:Rap guanine nucleotide exchange factor (GEF) 1, full insert sequence. Nucleotide: Bethesda (MD): National Library of Medicine (US), National Center for Biotechnology Information. https://www.ncbi.nlm.nih.gov/nuccore/AK147468.1 (2004).

[68] O'Leary NA (2016). Reference sequence (RefSeq) database at NCBI: current status, taxonomic expansion, and functional annotation. Nucleic Acids Res..

[69] AK147696.1. *Mus**musculus* cDNA, RIKEN full-length enriched library, clone:M5C1109D19 product:Rap guanine nucleotide exchange factor (GEF) 1, full insert sequence. Nucleotide: Bethesda (MD): National Library of Medicine (US), National Center for Biotechnology Information. https://www.ncbi.nlm.nih.gov/nuccore/AK147696.1 (2010).

[70] Nagayama S, Homma R, Imamura F (2014). Neuronal organization of olfactory bulb circuits. Front. Neural Circuits.

[71] Price JL, Powell TP (1970). The mitral and short axon cells of the olfactory bulb. J. Cell Sci..

[72] Kempermann G, Jessberger S, Steiner B, Kronenberg G (2004). Milestones of neuronal development in the adult hippocampus. Trends Neurosci..

[73] Elston GN (2003). Cortex, cognition and the cell: new insights into the pyramidal neuron and prefrontal function. Cereb. Cortex.

[74] Korotkova TM, Ponomarenko AA, Brown RE, Haas HL (2004). Functional diversity of ventral midbrain dopamine and GABAergic neurons. Mol. Neurobiol..

[75] Leuner B, Gould E (2010). Structural plasticity and hippocampal function. Annu. Rev. Psychol..

[76] Grove EA, Tole S (1999). Patterning events and specification signals in the developing hippocampus. Cereb. Cortex.

[77] Mullen RJ, Buck CR, Smith AM (1992). Neun, a neuronal specific nuclear-protein in vertebrates. Development.

[78] Urban N, Guillemot F (2015). Neurogenesis in the embryonic and adult brain: same regulators, different roles. Front. Cell Neurosci..

[79] Gertler FB, Bennett RL, Clark MJ, Hoffmann FM (1989). Drosophila abl tyrosine kinase in embryonic CNS axons: a role in axonogenesis is revealed through dosage-sensitive interactions with disabled. Cell.

[80] Kuo WL, Chung KC, Rosner MR (1997). Differentiation of central nervous system neuronal cells by fibroblast-derived growth factor requires at least two signaling pathways: roles for Ras and Src. Mol. Cell. Biol..

[81] Utreras E (2013). Cdk5 regulates Rap1 activity. Neurochem. Int..

[82] Wu DC (2000). The expression of Cdk5, p35, p39, and Cdk5 kinase activity in developing, adult, and aged rat brains. Neurochem. Res..

[83] Sievers F (2011). Fast, scalable generation of high-quality protein multiple sequence alignments using Clustal Omega. Mol. Syst. Biol..

[84] The UniProt C (2017). UniProt: the universal protein knowledgebase. Nucleic Acids Res..

[85] Li W (2015). The EMBL-EBI bioinformatics web and programmatic tools framework. Nucleic Acids Res..

[86] Altschul SF, Gish W, Miller W, Myers EW, Lipman DJ (1990). Basic local alignment search tool. J. Mol. Biol..

[87] Gutierrez-Berzal J (2006). Characterization of p87C3G, a novel, truncated C3G isoform that is overexpressed in chronic myeloid leukemia and interacts with Bcr-Abl. Exp. Cell Res..

[88] Blom N, Sicheritz-Ponten T, Gupta R, Gammeltoft S, Brunak S (2004). Prediction of post-translational glycosylation and phosphorylation of proteins from the amino acid sequence. Proteomics.

[89] Huttlin EL (2010). A tissue-specific atlas of mouse protein phosphorylation and expression. Cell.

[90] Sharma K (2015). Cell type- and brain region-resolved mouse brain proteome. Nat. Neurosci..

[91] Miya K (2008). Serine racemase is predominantly localized in neurons in mouse brain. J. Comp. Neurol..

[92] Omura T, Kaneko M, Tabei N, Okuma Y, Nomura Y (2008). Immunohistochemical localization of a ubiquitin ligase HRD1 in murine brain. J. Neurosci. Res..

[93] Huang CC, Lin YS, Lee CC, Hsu KS (2014). Cell type-specific expression of Eps8 in the mouse hippocampus. BMC Neurosci..

[94] Bayer SA, Altman J (1991). Development of the endopiriform nucleus and the claustrum in the rat brain. Neuroscience.

[95] Sun Y (2001). Neurogenin promotes neurogenesis and inhibits glial differentiation by independent mechanisms. Cell.

[96] Banelli B (2017). MicroRNA in glioblastoma: an overview. Int. J. Genomics.

[97] Sana J (2018). Identification of microRNAs differentially expressed in glioblastoma stem-like cells and their association with patient survival. Sci. Rep..

[98] Ramachandran S (2016). Cis-acting single nucleotide polymorphisms alter MicroRNA-mediated regulation of human brain-expressed transcripts. Hum. Mol. Genet..

[99] Zhu JJ, Qin Y, Zhao M, Van Aelst L, Malinow R (2002). Ras and Rap control AMPA receptor trafficking during synaptic plasticity. Cell.

[100] Wang N, Dhumale P, Chiang J, Puschel AW (2018). The Sema3A receptor Plexin-A1 suppresses supernumerary axons through Rap1 GTPases. Sci. Rep..

[101] Cahill ME (2016). Bidirectional synaptic structural plasticity after chronic cocaine administration occurs through Rap1 small GTPase signaling. Neuron.

[102] Shah B (2017). Rap1 GTPases are master regulators of neural cell polarity in the developing neocortex. Cereb. Cortex.

[103] Stupien G, Florian C, Roullet P (2003). Involvement of the hippocampal CA3-region in acquisition and in memory consolidation of spatial but not in object information in mice. Neurobiol. Learn. Mem..

[104] Susaimanickam PJ (2017). Generating minicorneal organoids from human induced pluripotent stem cells. Development.

[105] Yakoub AM, Sadek M (2018). Development and characterization of human cerebral organoids: an optimized protocol. Cell Transpl..

[106] Yamaguchi M (2005). Analysis of neurogenesis using transgenic mice expressing GFP with nestin gene regulatory regions. Chem. Senses.

[107] Goldring JPD, Ravaioli L (1997). Developing a dye-based method of protein quantitation on nitrocellulose. Biochem. Educ..

[108] Shevchenko A (1996). Linking genome and proteome by mass spectrometry: large-scale identification of yeast proteins from two dimensional gels. Proc. Natl. Acad. Sci. U.S.A..

[109] Rappsilber J, Friesen WJ, Paushkin S, Dreyfuss G, Mann M (2003). Detection of arginine dimethylated peptides by parallel precursor ion scanning mass spectrometry in positive ion mode. Anal. Chem..

