# Supplementary material for: Expression of a novel brain specific isoform of C3G is regulated during development
Source: Sci Rep. 2020 Nov 2;10:18838. doi: 10.1038/s41598-020-75813-z (PMC7606606; doi:10.1038/s41598-020-75813-z)
Supplement: Supplementary file 1 — Supplementary Figures. [file 41598_2020_75813_MOESM1_ESM.pdf]

TITLE: **Expression of a novel brain specific isoform of C3G is regulated during development**

AUTHORS: Divya Sriram[#1,] Ramulu Chintala[#1], B.V.V. Parthasaradhi[1], Sanjeev Chavan Nayak[1], Indumathi Mariappan[2], Vegesna Radha[1]*

# equal contribution

AFFILIATION: [1]CSIR-Centre for Cellular and Molecular Biology
Uppal Road
Hyderabad – 500 007
INDIA
[2] Sudhakar and Sreekanth Ravi Stem Cell Biology Laboratory, Prof Brien Holden Eye Research Centre, Hyderabad Eye Research Foundation,
L.V. Prasad Eye Institute,
Hyderabad, INDIA

Running Title: **Brain specific C3G isoform**

***CORRESPONDING AUTHOR:**
VEGESNA RADHA
Chief Scientist
CSIR-Centre for Cellular and Molecular Biology
Uppal Road
Hyderabad – 500 007
INDIA
Tel: +91-40-27192619 / +91-40-27160222
Fax: +91-40-27160591 / +91-40-27160311
e.mail: vradha@ccmb.res.in

Supplementary figure 1

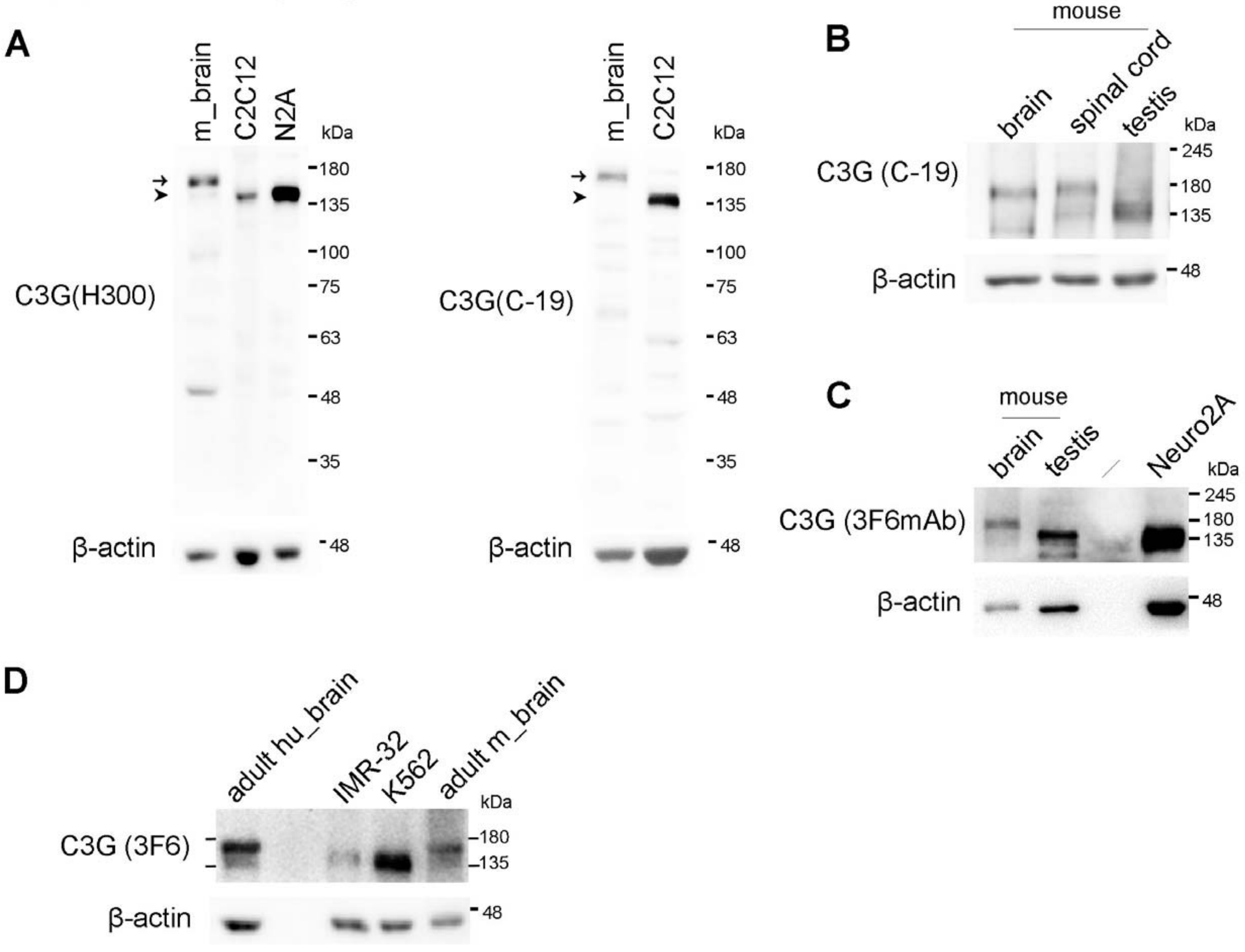

Supplementary figure 1. Validation of antibody specificity and expression of C3G in mouse brain. A) Western blots showing specificity of two C3G antibodies, H-300 and C-19, that were raised against N-ter, and C-ter sequences of C3G, respectively. Larger polypeptides seen in brain are indicated by arrow, and the previously identified 140 kDa form indicated by an arrowhead. **B)** Expression of C3G isoforms in brain, spinal cord and testis as checked by western blotting using C3G C-19 antibody. **C)** Expression of C3G isoforms in lysates of mouse brain, testis and Neuro2A cells was checked by western blotting using C3G 3F6mAb. D) Expression of C3G in human brain tissue. Lysates from human brain, IMR-32 cells, K562 cells and 1-yr-mouse brain tissue were subjected to western blotting and probed for C3G (3F6). β-actin was used as a loading control.

Supplementary figure 2

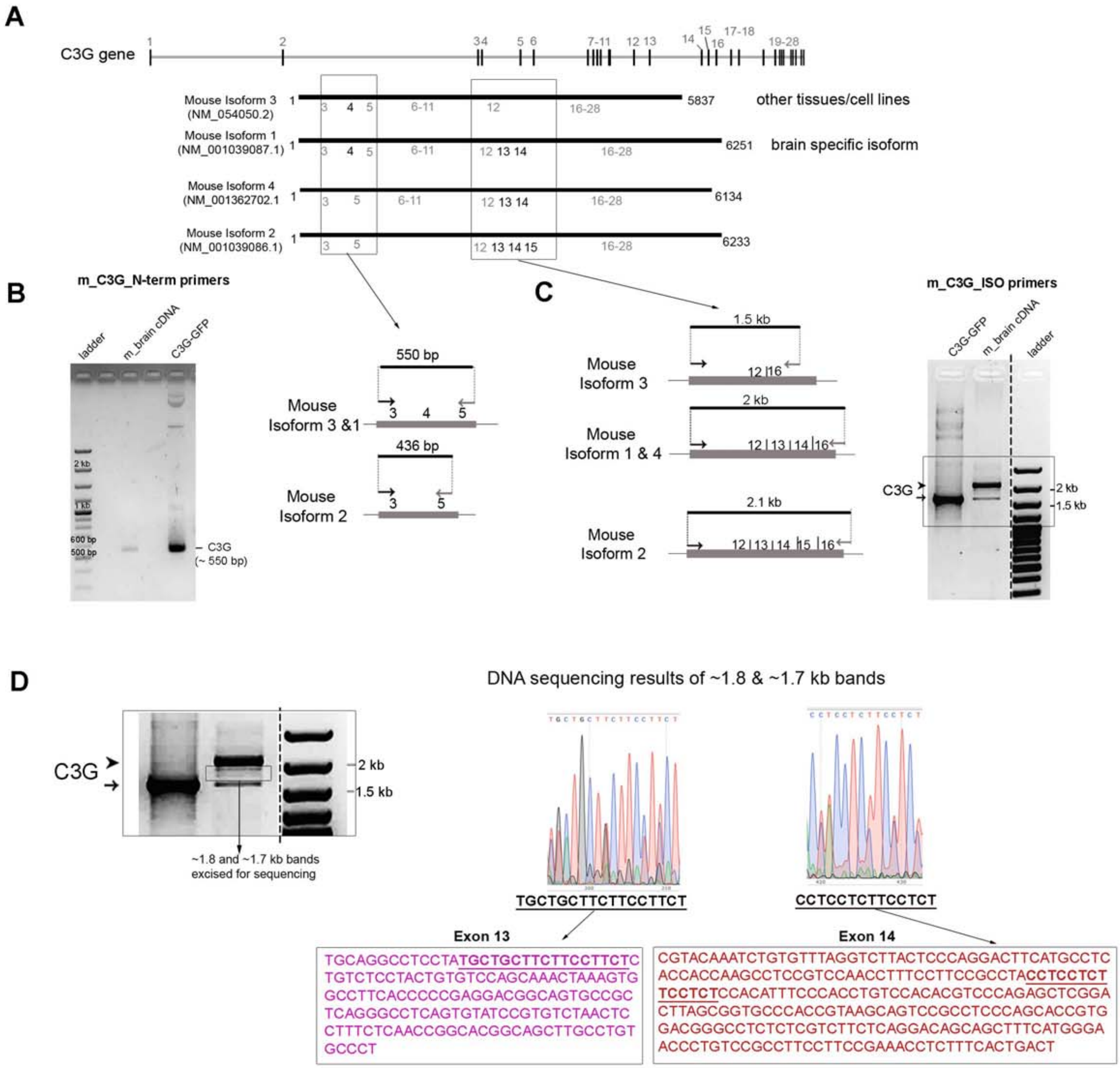

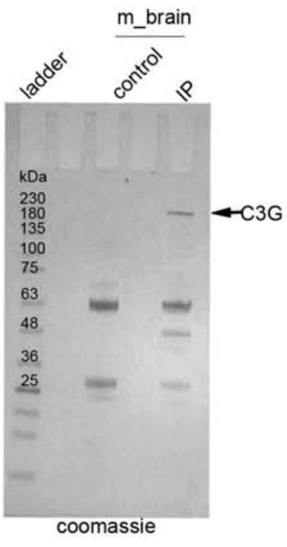

Supplementary figure 2. Identification of mouse brain C3G isoform by isoform specific PCR and sequencing. A) Schematic representation of mouse C3G gene in mouse and isoforms arising from alternate splicing are shown below with numbers indicating the presence of specific exons. Based on size of protein, longer brain isoform could correspond to Isoforms 1 & 4. **B)** RNA was isolated form mouse brain and cDNA prepared was subjected to PCR using m_C3G N-ter primers that would form PCR products of different lengths based on presence or absence of exon 4. It was confirmed that the mouse brain C3G isoform has exon 4, thus cannot be Isoform 2 & 4 (which lack exon 4). Agarose gel is shown in the left with a schematic representation of possible PCR products (and their expected sizes) are shown in the right. The arrows indicate forward and reverse primers. **C)** Mouse cDNA was subjected to PCR using m_C3G_ISO primers that would give specific products of given lengths based on presence of exons 12,13,14 and 15. The prominent 2 kb product indicates that the C3G brain isoform is Isoform 1 (further details in Suppl.fig.3). C3G-GFP plasmid (corresonding to human isoform a) showing product size 1.5 kb was used as a reference. Agarose gel is shown in the right side, while left side shows a schematic represntation of possible PCR products from each isoform and their expectd sizes. Arrows in the schematic represent the forward and reverse primers. Arrowhead and arrow in agarose gel, point to the 2kb and 1.5 kb bands, respectively. **D)** To identify the weakly expressed 1.7kb and 1.8 kb products they were excised from gel in C, (as shown in the box) and sequenced. They were found to contain either Exon 13 or Exon 14 but not Exon 15. Exon 13 and 14 are shown in boxes for reference and the matched sequences underlined. **E)** C3G was immunoprecipitated from mouse brain using C3G (G-4) antibody (IP) or mIgG (control). They were subjected to SDS-PAGE and the gel was stained with coomassie. Image shows the coomassie stained gel containing a clean band around ~175 kDa, that corresponds to the size of C3G in mouse brain (which is absent in the IgG control lane). This ~175 kDa band was excised and processed for mass spectrometry.

Supplementary figure 3

A

DNA Sequencing of 2kb product

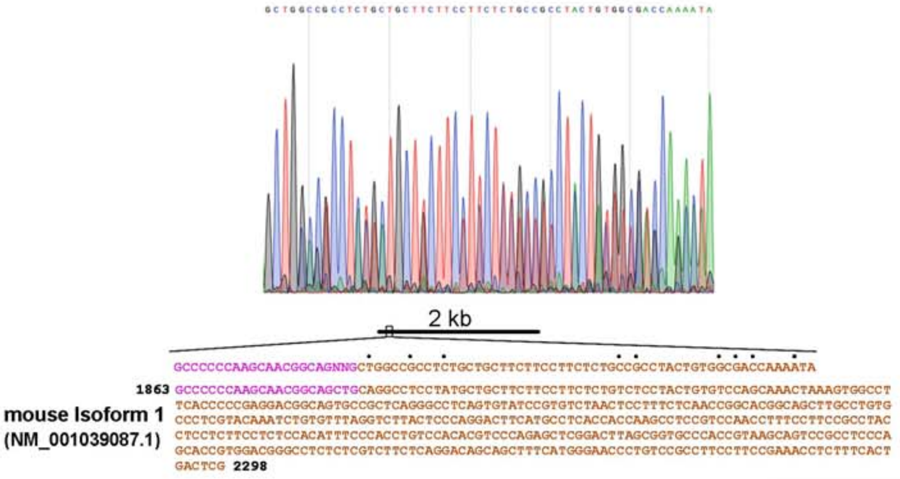

BLASTN RESULT

Description	Max Score	Total Score	Query Cover	E value	Per. Ident	Accession
PREDICTED: Mus musculus Rap guanine nucleotide exchange factor (GEF) 1 (Rapgef1), transcript variant X8, mRNA	92.4	92.4	100%	5e-18	85.71%	XM_006497589.3
PREDICTED: Mus musculus Rap guanine nucleotide exchange factor (GEF) 1 (Rapgef1), transcript variant X5, mRNA	92.4	92.4	100%	5e-18	85.71%	XM_006497586.3
PREDICTED: Mus musculus Rap guanine nucleotide exchange factor (GEF) 1 (Rapgef1), transcript variant X4, mRNA	92.4	92.4	100%	5e-18	85.71%	XM_006497585.3
PREDICTED: Mus musculus Rap guanine nucleotide exchange factor (GEF) 1 (Rapgef1), transcript variant X1, mRNA	92.4	92.4	100%	5e-18	85.71%	XM_006497582.3
PREDICTED: Mus musculus Rap guanine nucleotide exchange factor (GEF) 1 (Rapgef1), transcript variant X2, mRNA	92.4	92.4	100%	5e-18	85.71%	XM_006497583.1
# Mus musculus Rap guanine nucleotide exchange factor (GEF) 1 (Rapgef1), transcript variant 1, mRNA	92.4	92.4	100%	5e-18	85.71%	NM_001039087.1
# Mus musculus Rap guanine nucleotide exchange factor (GEF) 1 (Rapgef1), transcript variant 2, mRNA	92.4	92.4	100%	5e-18	85.71%	NM_001039086.1
# Mus musculus Rap guanine nucleotide exchange factor (GEF) 1 (Rapgef1), transcript variant 4, mRNA	88.7	88.7	100%	7e-17	85.71%	NM_001362702.1
PREDICTED: Mus musculus Rap guanine nucleotide exchange factor (GEF) 1 (Rapgef1), transcript variant X9, mRNA	88.7	88.7	100%	7e-17	85.71%	XM_006497590.3
PREDICTED: Mus musculus Rap guanine nucleotide exchange factor (GEF) 1 (Rapgef1), transcript variant X3, mRNA	88.7	88.7	100%	7e-17	85.71%	XM_006497584.3

#

Description	Max Score	Total Score	Query Cover	E value	Per. Ident	Accession
Mus musculus Rap guanine nucleotide exchange factor (GEF) 1 (Rapgef1), transcript variant 1, mRNA	92.4	92.4	100%	5e-18	85.71%	NM_001039087.1
Mus musculus Rap guanine nucleotide exchange factor (GEF) 1 (Rapgef1), transcript variant 2, mRNA	92.4	92.4	100%	5e-18	85.71%	NM_001039086.1
Mus musculus Rap guanine nucleotide exchange factor (GEF) 1 (Rapgef1), transcript variant 4, mRNA	88.7	88.7	100%	7e-17	85.71%	NM_001362702.1

B

DNA Sequencing of 1.5kb product

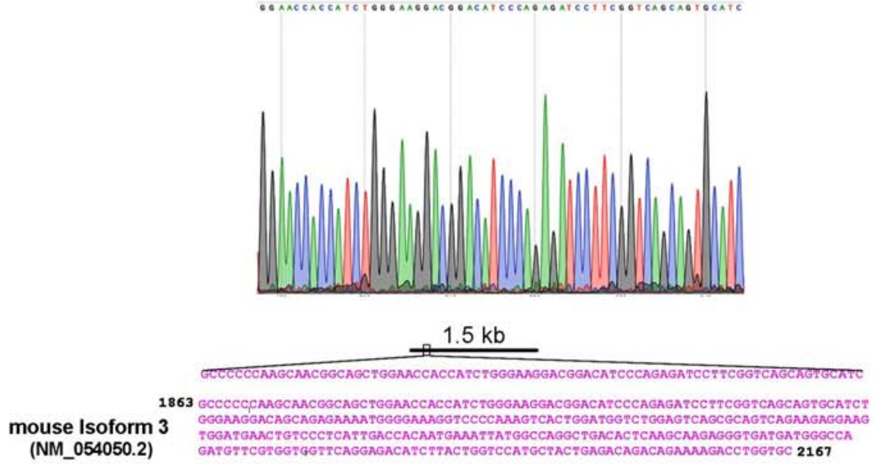

BLASTN RESULT

Description	Max Score	Total Score	Query Cover	E value	Per. Ident	Accession
Transcripts						
PREDICTED: Mus musculus Rap guanine nucleotide exchange factor (GEF) 1 (Rapgef1), transcript variant X10, mRNA	145	145	100%	3e-33	100.00%	XM_006497592.3
* Mus musculus Rap guanine nucleotide exchange factor (GEF) 1 (Rapgef1), transcript variant 3, mRNA	145	145	100%	3e-33	100.00%	NM_054050.2
PREDICTED: Mus musculus Rap guanine nucleotide exchange factor (GEF) 1 (Rapgef1), transcript variant X9, mRNA	108	108	74%	4e-22	100.00%	XM_006497590.3
PREDICTED: Mus musculus Rap guanine nucleotide exchange factor (GEF) 1 (Rapgef1), transcript variant X8, mRNA	108	108	74%	4e-22	100.00%	XM_006497589.3
PREDICTED: Mus musculus Rap guanine nucleotide exchange factor (GEF) 1 (Rapgef1), transcript variant X4, mRNA	108	108	74%	4e-22	100.00%	XM_006497585.3
PREDICTED: Mus musculus Rap guanine nucleotide exchange factor (GEF) 1 (Rapgef1), transcript variant X7, mRNA	108	108	74%	4e-22	100.00%	XM_006497588.1
Mus musculus Rap guanine nucleotide exchange factor (GEF) 1 (Rapgef1), transcript variant 1, mRNA	108	108	74%	4e-22	100.00%	NM_001039087.1
PREDICTED: Mus musculus Rap guanine nucleotide exchange factor (GEF) 1 (Rapgef1), transcript variant X6, mRNA	106	106	73%	2e-21	100.00%	XM_006497587.3
PREDICTED: Mus musculus Rap guanine nucleotide exchange factor (GEF) 1 (Rapgef1), transcript variant X5, mRNA	106	106	73%	2e-21	100.00%	XM_006497586.3
PREDICTED: Mus musculus Rap guanine nucleotide exchange factor (GEF) 1 (Rapgef1), transcript variant X3, mRNA	106	106	73%	2e-21	100.00%	XM_006497584.3
PREDICTED: Mus musculus Rap guanine nucleotide exchange factor (GEF) 1 (Rapgef1), transcript variant X1, mRNA	106	106	73%	2e-21	100.00%	XM_006497582.3
PREDICTED: Mus musculus Rap guanine nucleotide exchange factor (GEF) 1 (Rapgef1), transcript variant X2, mRNA	106	106	73%	2e-21	100.00%	XM_006497583.1
Mus musculus Rap guanine nucleotide exchange factor (GEF) 1 (Rapgef1), transcript variant 2, mRNA	106	106	73%	2e-21	100.00%	NM_001039086.1
Genomic sequences[show first]						
Mus musculus strain C57BL/6J chromosome 2, GRCm38.p4 C57BL/6J	108	108	78%	4e-22	98.36%	NC_000068.7

Description	Max Score	Total Score	Query Cover	E value	Per. Ident	Accession
* Mus musculus Rap guanine nucleotide exchange factor (GEF) 1 (Rapgef1), transcript variant 3, mRNA	145	145	100%	3e-33	100.00%	NM_054050.2

Supplementary figure 3. DNA Sequencing of mouse C3G amplicons. The 2 kb and 1.5 kb DNA bands obtained by PCR amplification were separately excised from the agarose gel, purified and subjected to sequencing using the C3G_ISO-F primer. **A)** Chromatogram of DNA sequencing shows a stretch of 56 nucleotides sequenced from the 2 kb DNA fragment (expanded below). Sequence was subjected to nucleotide BLAST (BLASTN, an NCBI search tool) against mouse genome and transcriptome consortium in NCBI. The BLAST result is shown and the box highlights the isoforms detected. Sequences in mouse Isoform 1 (NM_001039087.1) are shown below for comparison. The mismatches shown as dots may be due to PCR/sequencing errors. **B)** Chromatogram shows a stretch of 78 nucleotides sequenced from the 1.5 kb DNA fragment (expanded below). This sequence matches mouse Isoform 3 (NM_054050.2) and is shown below. Nucleotides highlighted in pink match sequences in NM_054050.2, while sequences unique to NM_001039087.1 are highlighted as brown.

Supplementary figure 4

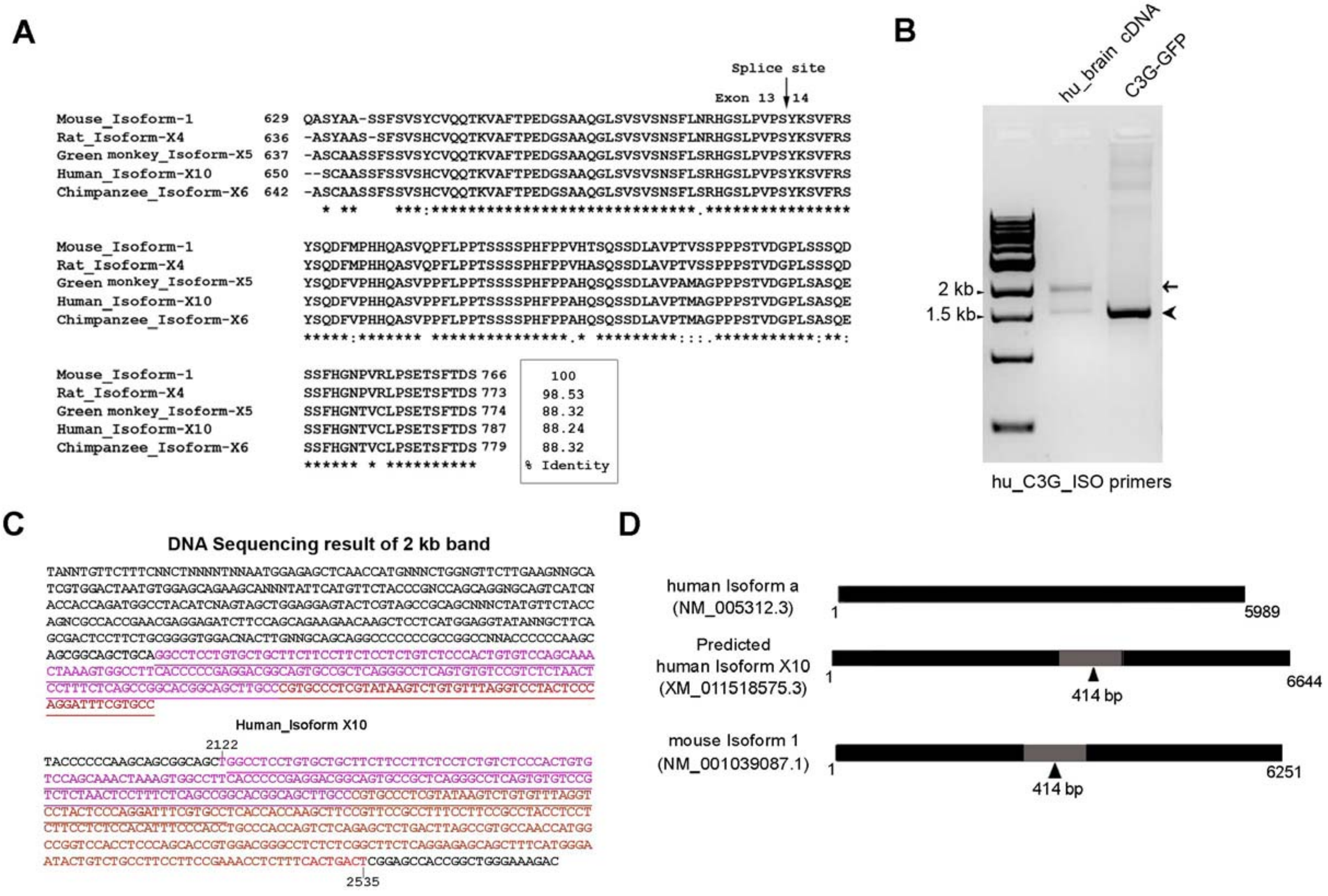

Supplementary figure 4. Identification of human brain C3G isoform. A) Conservation of protein sequences corresponding to exons 13 and 14, among indicated species in comparison to mouse isoform 1, using Clustal Omega. Exons 13 &14 are highly conserved among these species. **B)** Human brain RNA was extracted and prepared cDNA subjected to PCR using hu_C3G_ISO primers that will amplify products of different sizes based on presence or absence of exons in central C3G. C3G-GFP plasmid (corresonding to human isoform a) was used as a reference. Two products, the prominent 2 kb and 1.5 kb were amplified from human brain cDNA, indicating presence of extra exons in the 2 kb product compared to 1.5kb one. C3G-GFP showed only the 1.5 kb band. **C)** Sequencing of the 2 kb product revealed presence of exons that matched the predicted human isoform X10. Pink and red indicate matched sequences of exons 13 and 14, respectively, from mouse C3G isoform 1. **D)** Schematic representation of cDNA of human C3G isoforms a, predicted isoform X10 and mouse isoform 1. Predicted human isoform X10 contains 414 bp extra, similar to mouse isoform 1 (exons 13 & 14). The human isoform a, lacks these exons.

Supplementary figure 5

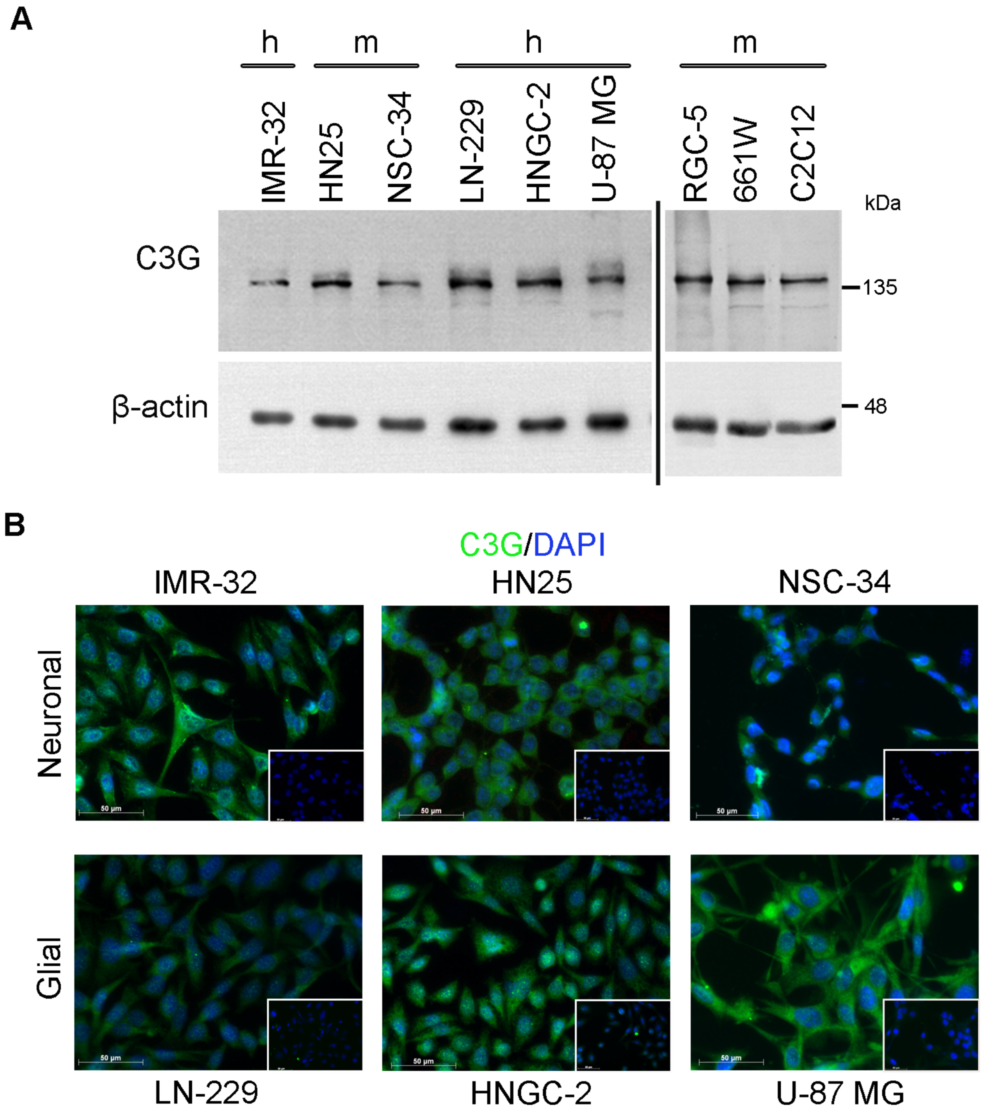

Supplementary figure 5. C3G expression in neuronal and glial cell lines of murine (m) and human-origin (h). A) Lysates of HN25 (neuronal, m), NSC-34 (neuronal, m), RGC-5 (neuronal, m), 661W (neuronal, m), C2C12 (myoblast, m), IMR-32 (neuronal, h), LN229 (glial, h), HNGC2 (glial, h) and U87-MG (glial, h) cells were subjected to western blotting and probed with H-300 antibody to detect C3G. β-actin was used as loading control. Vertical line separates two different blots that were processed and exposed at the same time under similar conditions. **B)** HN25, NSC-34, IMR-32, LN-229, HNGC-2 and U-87 MG cells were plated on coverslips, fixed and stained for C3G (green). Insert images are blanks of the same magnification. Scale bar, 50μm.

Supplementary figure 6

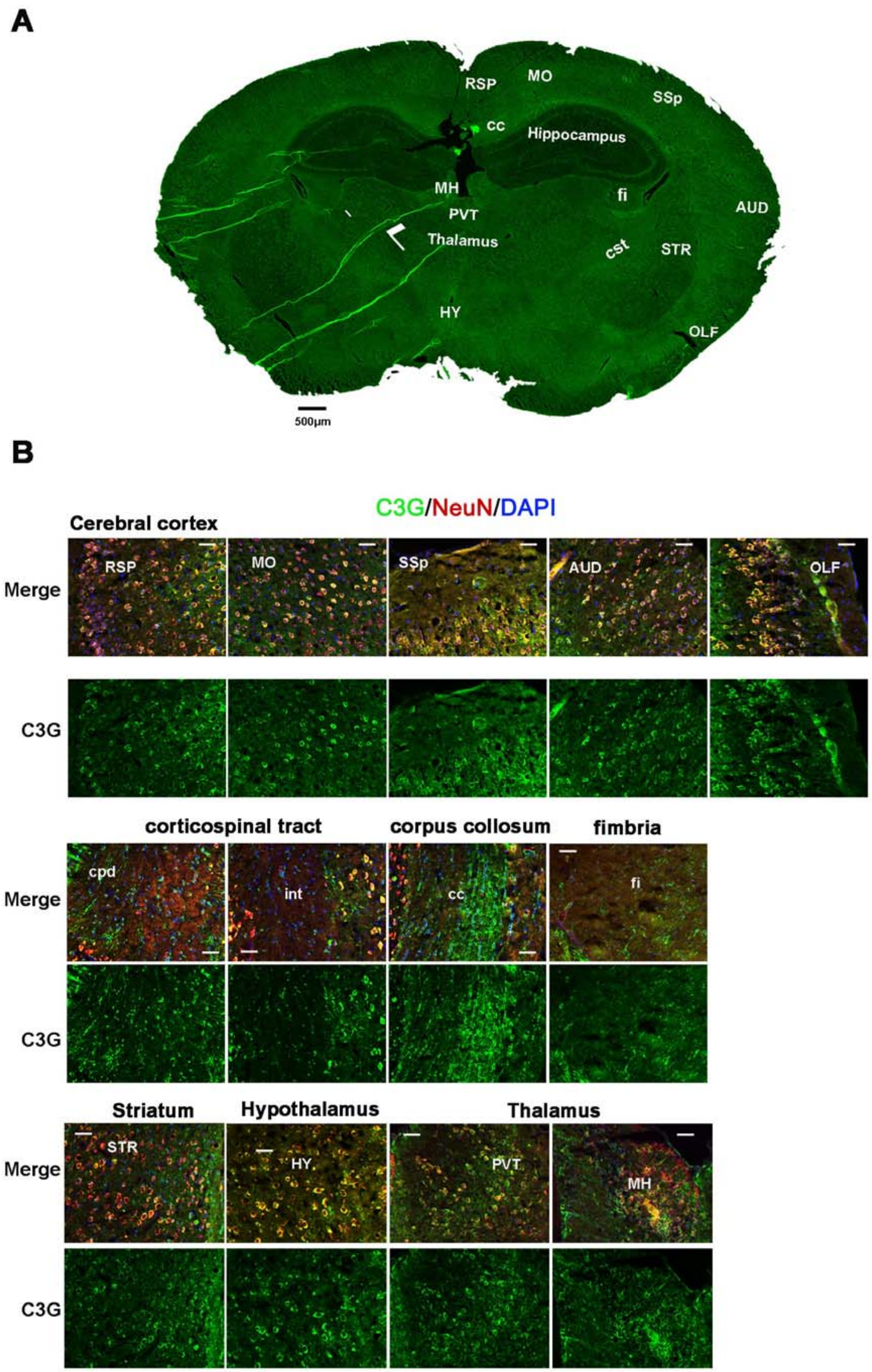

Supplementary figure 6. C3G expression in mid-brain regions of 3 months-old mouse. A) Reconstructed image of mid-brain regions after staining for C3G expression. Scale bar, 500µm.**B)** Images of different regions of mid-brain stained with C3G (H-300) (green) and NeuN (red) captured using 20X objective are shown along with corresponding C3G staining alone. (RSP, Retrosplenial area; MO, Somatomotor areas; SSp, Primary somatosensory area; AUD, Auditory areas; OLF, Olfactory areas; cc, corpus callosum; fi, fimbria; cst, corticospinal tract; cpd, cerebral peduncle; int, internal capsule; MH, medial habenula; PVT, paraventricular nucleus of the thalamus; STR, Striatum; HY, Hypothalamus). Images were taken using Axioimager Z1 microscope. Scale bar, 50µm.

Supplementary figure 7

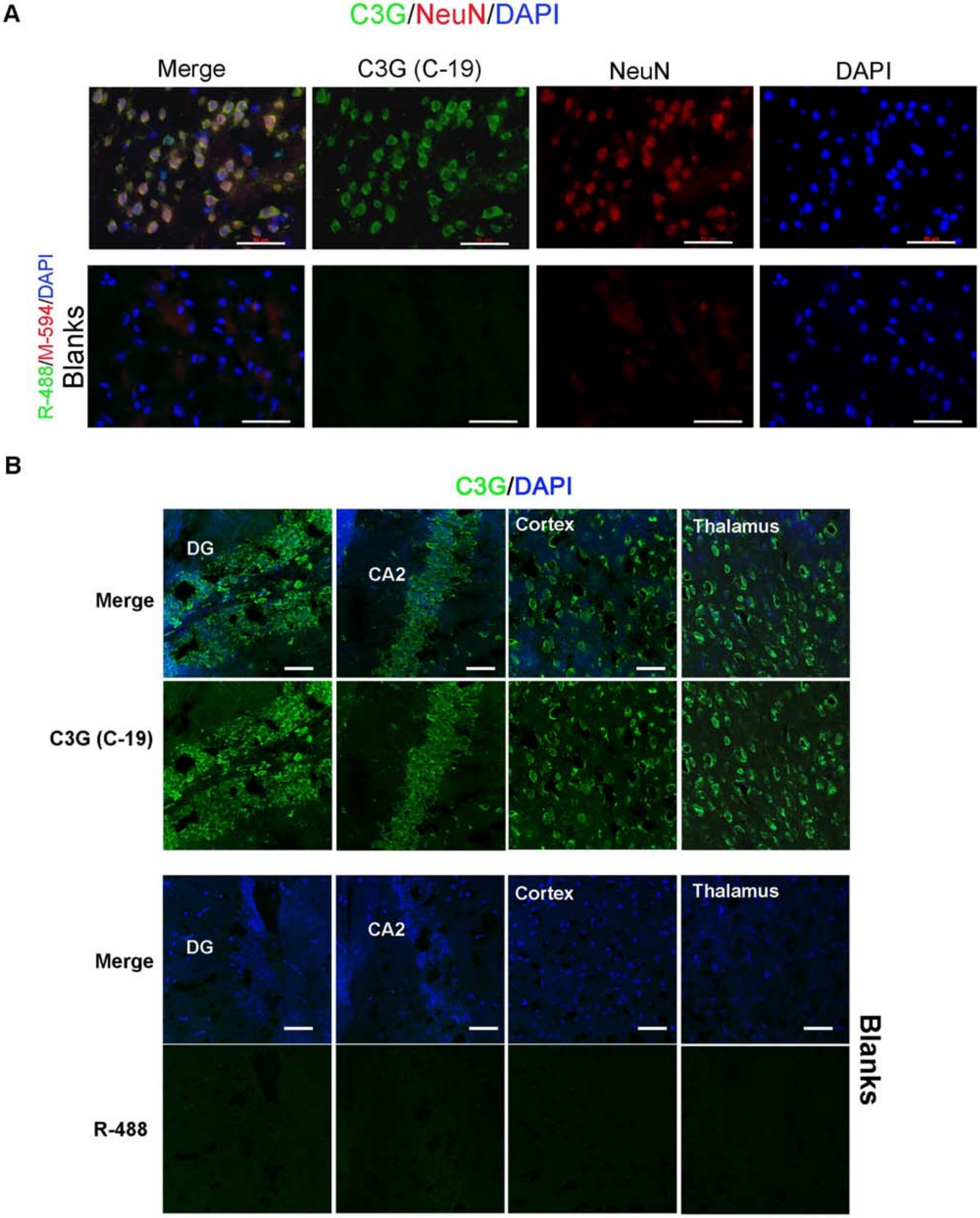

Supplementary figure 7. (A) C3G expression in cortical neurons. Sections from mouse brain cortex were stained for C3G using an antibody targeting its C-terminus (C-19). All neurons, identified by co-staining for NeuN, showed C3G predominantly in the cytoplasm. Blanks show sections processed similarly, but without addition of primary antibodies. Images were captured using Axioimager Z1, Carl Zeiss microscope. Panels show background signals arising from secondary antibodies anti-rabbit Alexa-488 (R-488) and anti-mouse Alexa-594 (M-594). Scale bar, 50µm. **(B) C3G expression in cortex, thalamus and hippocampal regions.** Sections from mouse brain cortex, thalamus and hippocampus (DG, CA2) were stained for C3G using C-19 antibody. Blanks show sections processed similarly by incubating with anti-rabbit Alexa-488 (R-488) secondary antibody, without the addition of C3G primary antibody. Images were captured using Leica TCS SP8 confocal microscope. Scale bar, 50µm.

Supplementary figure 8

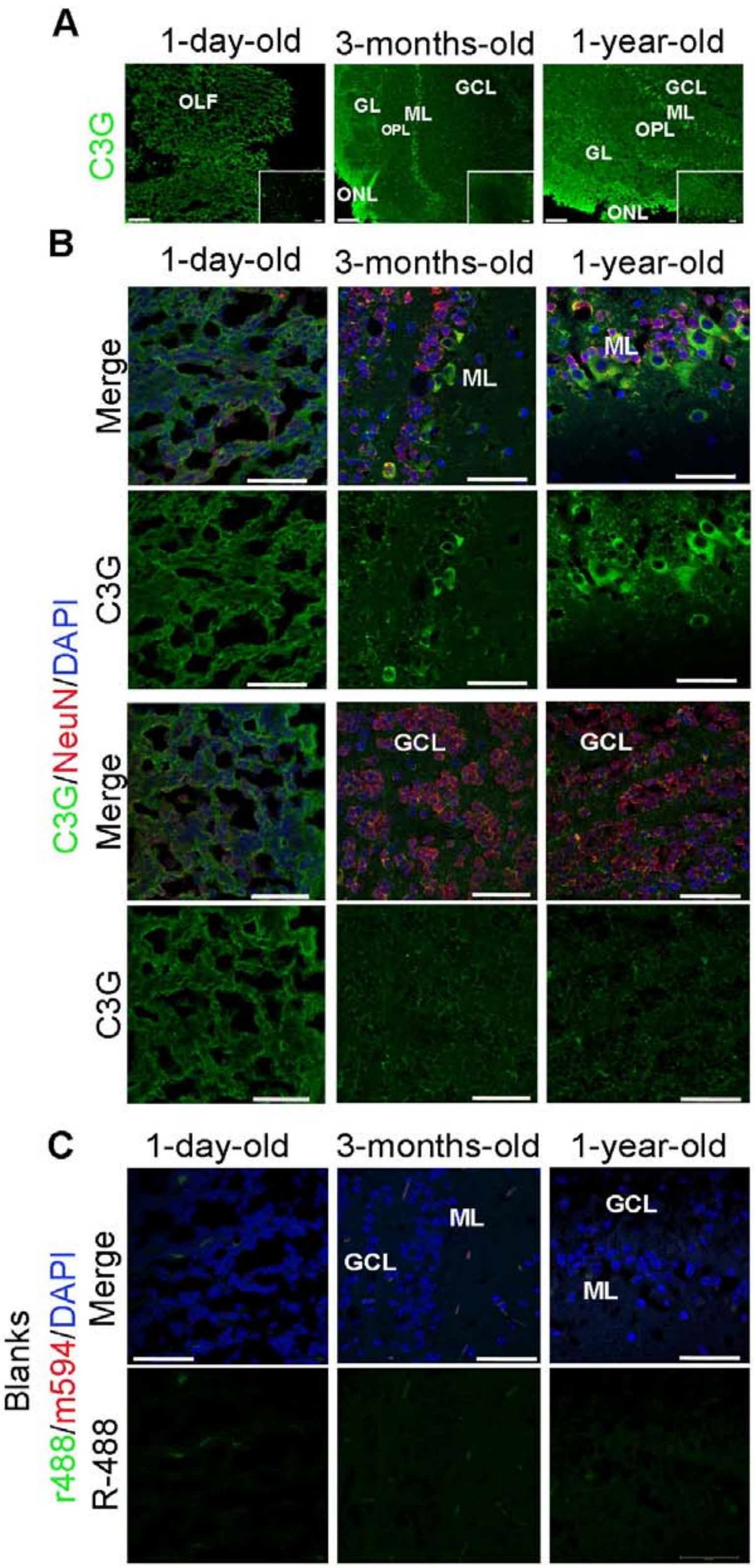

Supplementary figure 8. C3G expression in olfactory bulbs of mice of different age groups. A) Low magnification images of C3G stained sections obtained using Axioimager Z1. Scale bar, 100µm. **(B)** Higher magnification (63X) confocal images of olfactory bulb of 1-day-old, 3-months-old and 1-year-old mice stained with C3G (H-300) (green) and NeuN (red). Panels showing corresponding C3G staining are shown below. Scale bar, 50µm. **C)** Blank images show adjoining sections processed similarly without addition of primary anti-bodies. Secondary antibodies anti-rabbit Alexa-488 (R-488) and anti-mouse Alexa-594 (M-594) were used. Scale bar, 50µm. Different regions are indicated as OLF, Olfactory areas; ONL, olfactory nerve layer; OPL, outer plexiform layer; ML, mitral cell layer; GCL, granule cell layer.

Supplementary figure 9

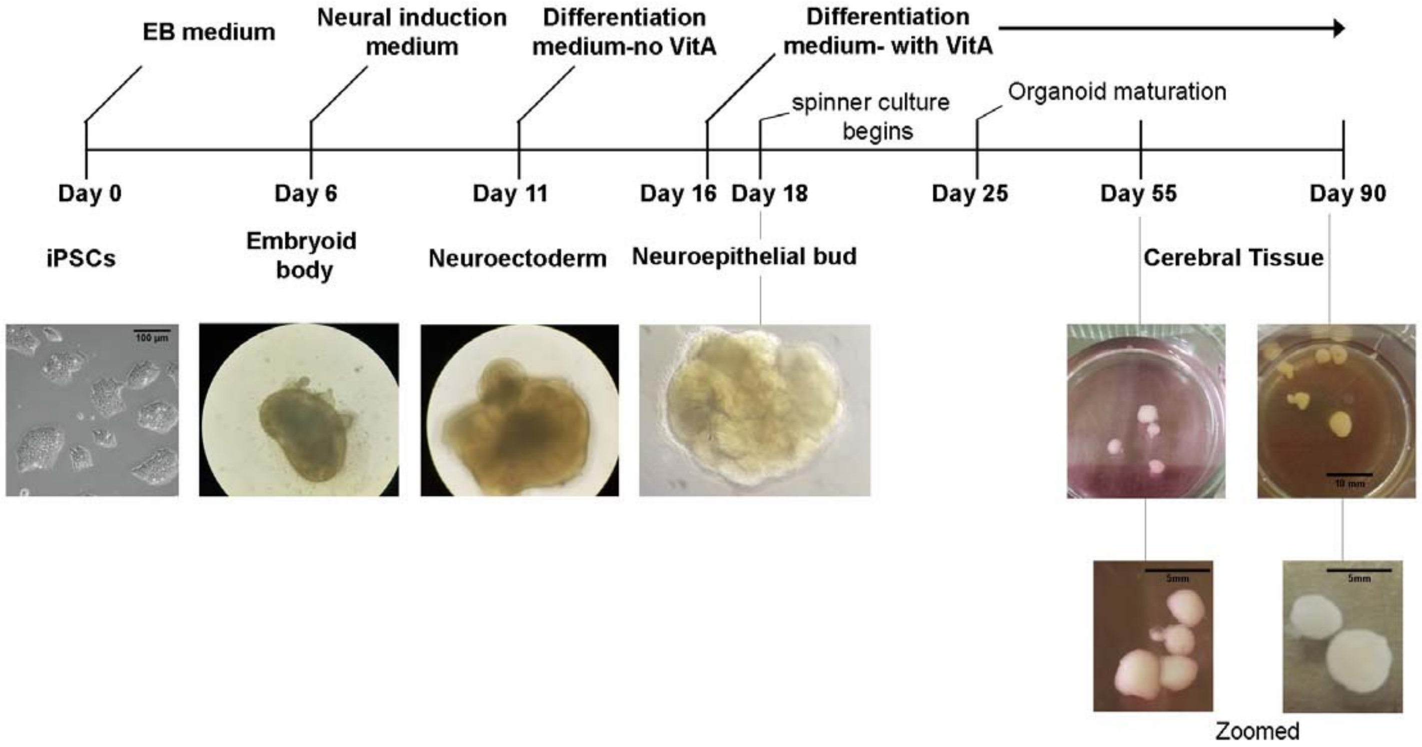

Supplementary figure 9. Differentiation of human cerebral organoids from iPSCs. The protocol (Yakoub & Sadek 2018) followed for human cerebral organoid development is schematically represented. Images depict various stages of growth from iPSCs.

Supplementary Information-Original blots

The images of the nitrocellulose membrane, used for probing, and showing the mol.wt.markers, are aligned with the immunoblots

Figure 1A

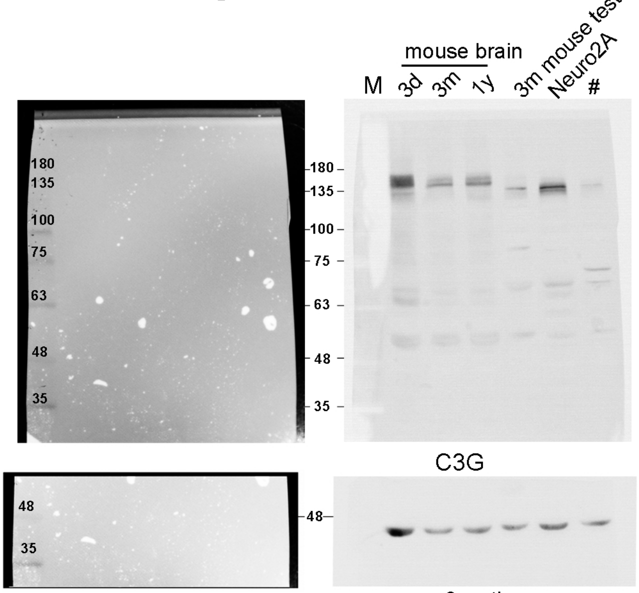

Figure 1B

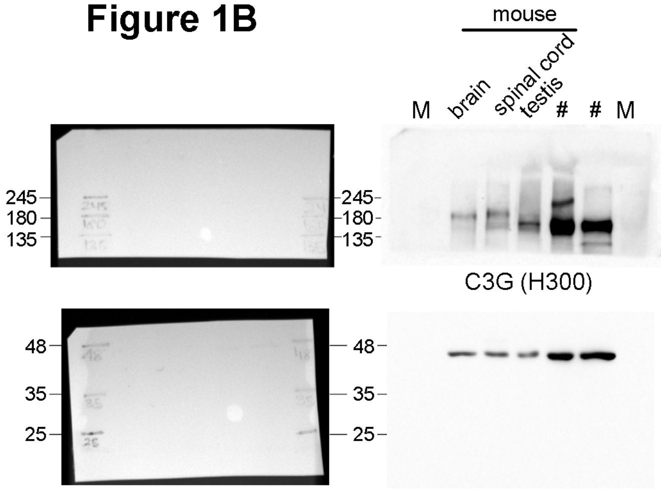

Supplementary figure 1B

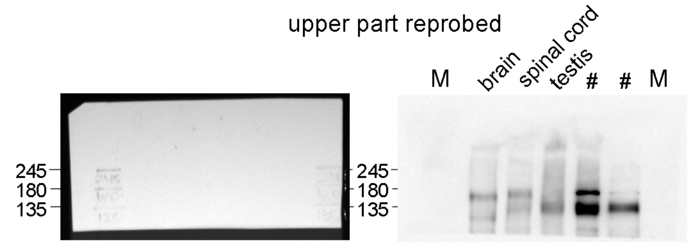

Figure 1D

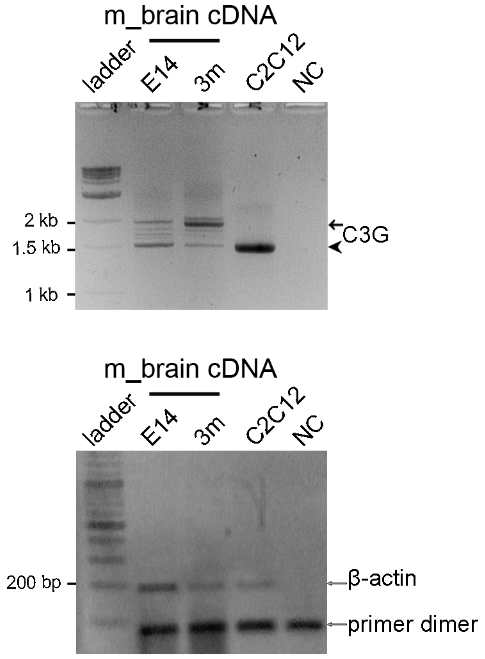

Figure 1F

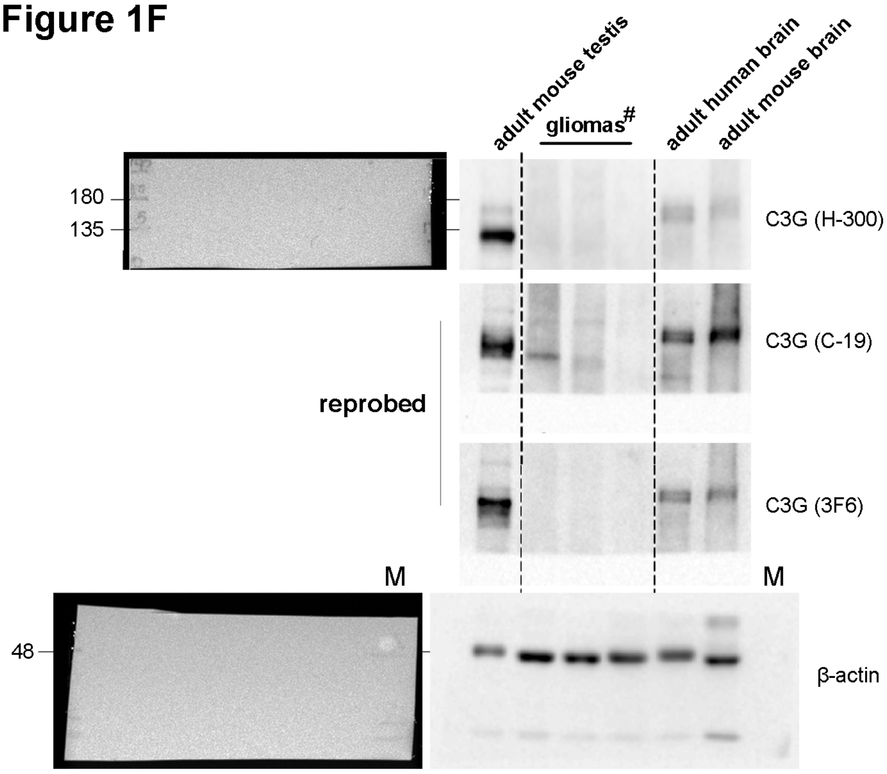

M - protein molecular weight marker

# These lanes are not used in the corresponding figures presented in the manuscript.

Supplementary information-Original blots

Supplementary figure 1A

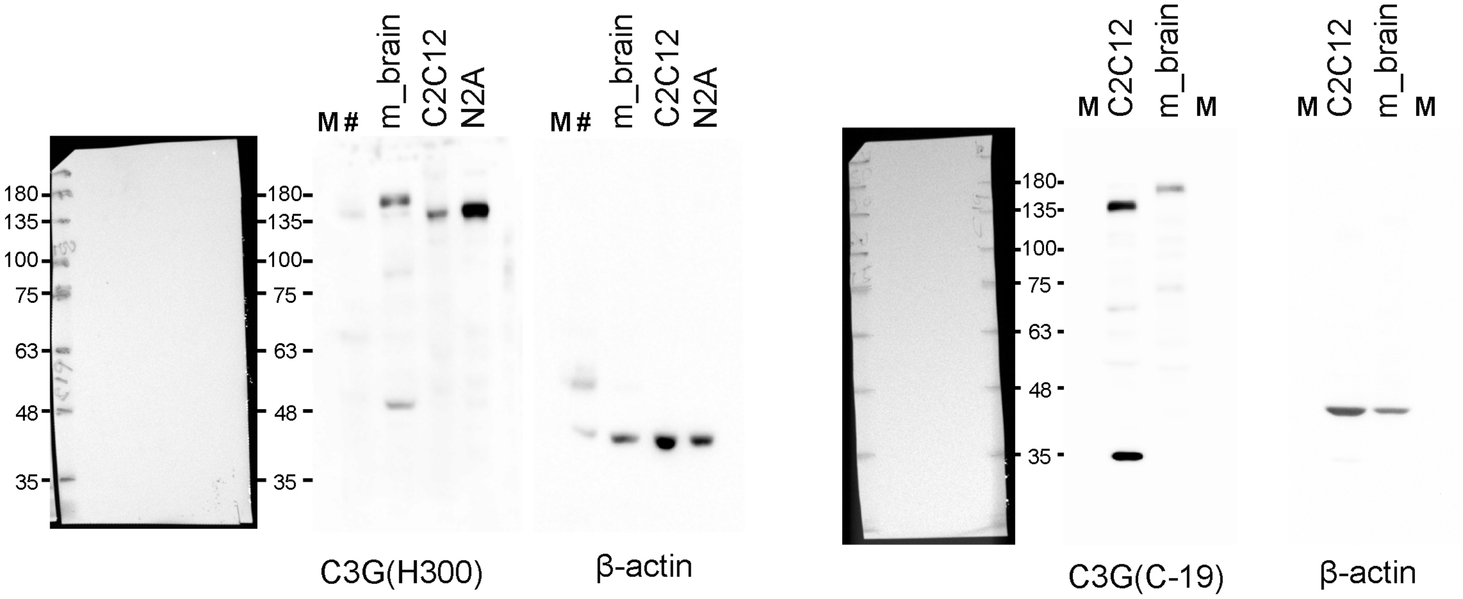

C-19 blots were flipped and shown in manuscript

Supplementary figure 1C

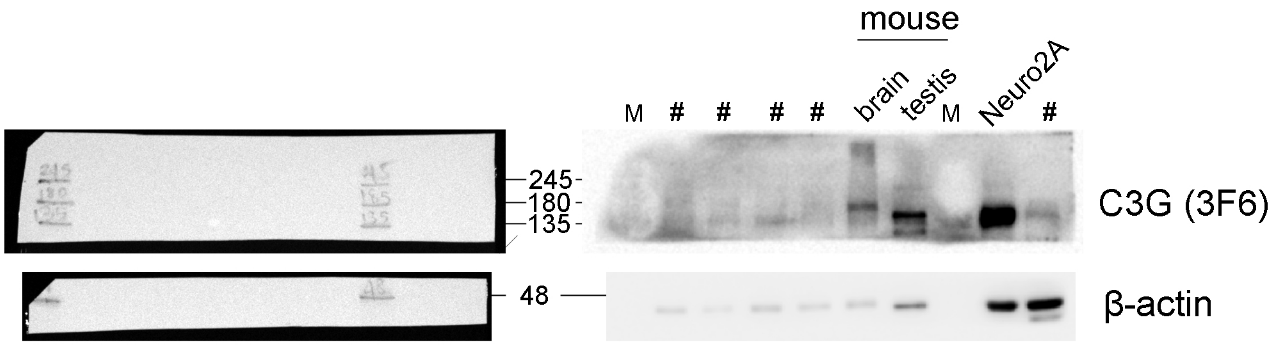

Supplementary figure 1D

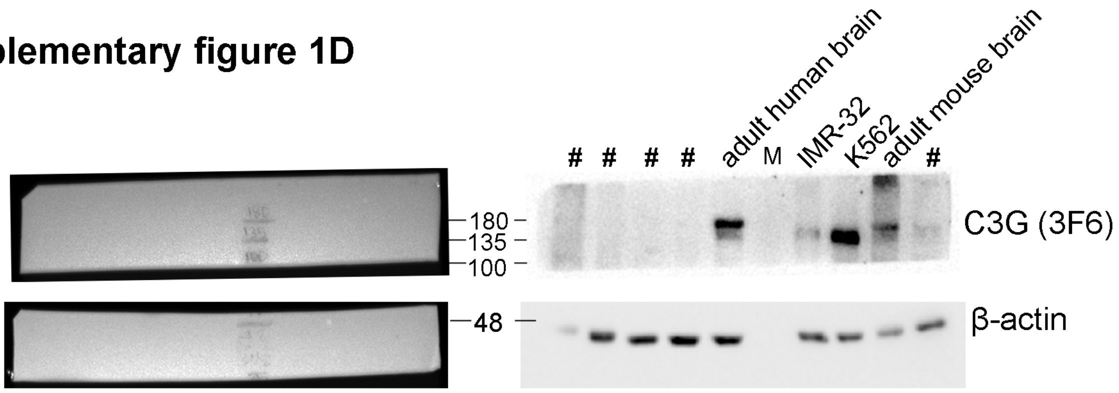

M - protein molecular weight marker

# These lanes are not used in the corresponding figures presented in the manuscript.

Supplementary information-Original blots

Figure 5A

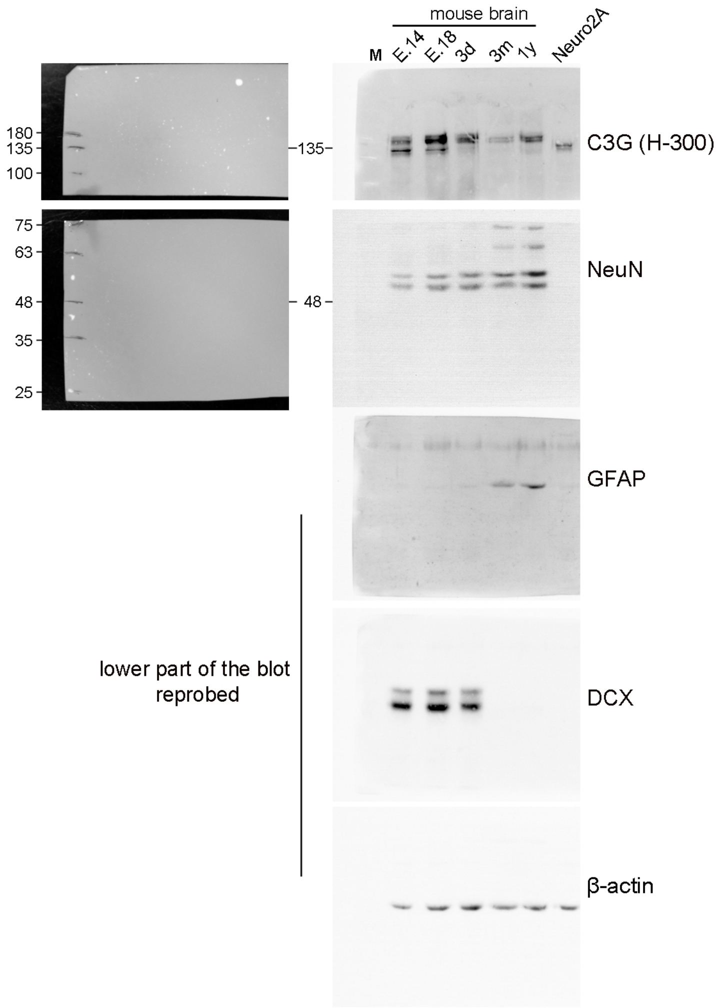

Figure 5B

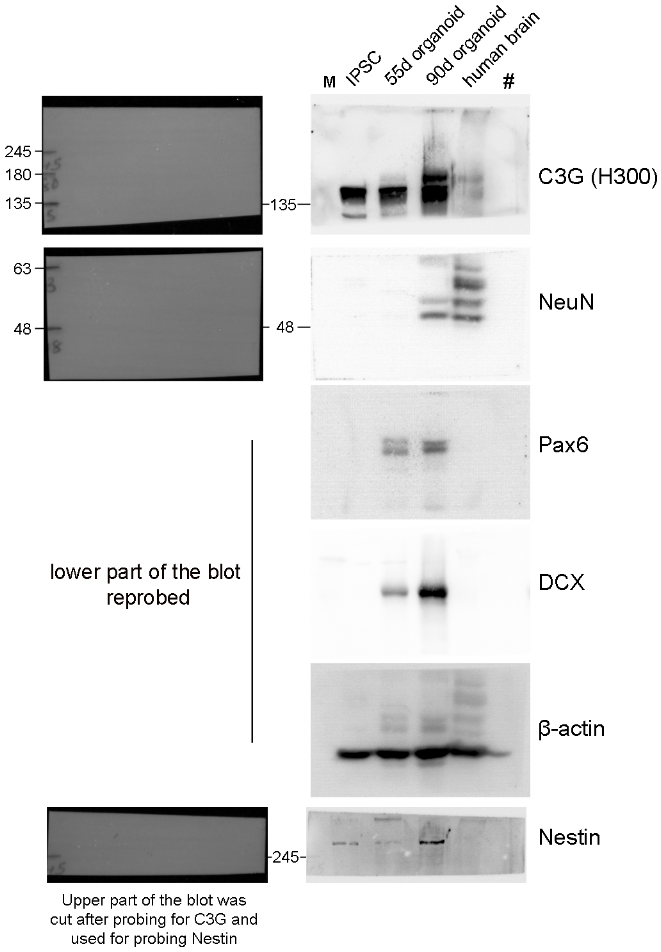

Supplementary figure 5A

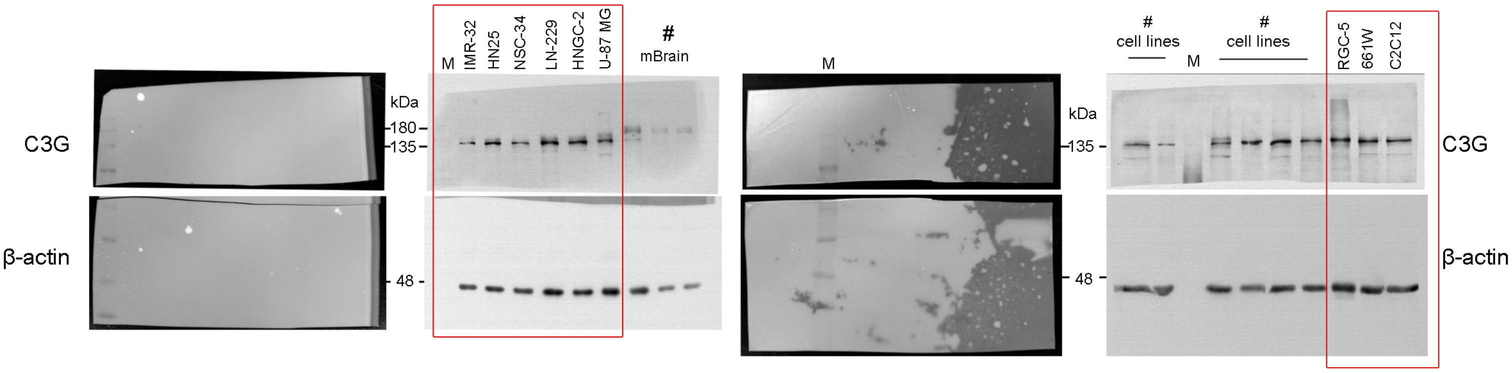

Lanes within the red box from the above two blots were used to make the figure

M - protein molecular weight marker

# These lanes are not used in the corresponding figures presented in the manuscript.
